# Mitochondrial, metabolic and bioenergetic adaptations drive plasticity of colorectal cancer cells and shape their chemosensitivity

**DOI:** 10.1038/s41419-025-07596-y

**Published:** 2025-04-05

**Authors:** Nikita Markov, Sirina Sabirova, Gulnaz Sharapova, Marina Gomzikova, Anna Brichkina, Nick A. Barlev, Marcel Egger, Albert Rizvanov, Hans-Uwe Simon

**Affiliations:** 1 Institute of Pharmacology, University of Bern, Bern, Switzerland; 2 Laboratory of Molecular Immunology, Institute of Fundamental Medicine and Biology, Kazan Federal University, Kazan, Russia; 3 Laboratory of Intercellular Communication, Kazan Federal University, Kazan, Russia; 4 OpenLab Gene and Cell Technology, Institute of Fundamental Medicine and Biology, Kazan Federal University, Kazan, Russia; 5 Institute of Systems Immunology, Center for Tumor Biology and Immunology, Philipps University of Marburg, Marburg, Germany; 6 Institute of Cytology of the Russian Academy of Sciences, St. Petersburg, Russia; 7 Department of Biomedical Sciences, School of Medicine, Nazarbayev University, Astana, Kazakhstan; 8 Department of Physiology, University of Bern, Bern, Switzerland; 9 Division of Medical and Biological Sciences, Tatarstan Academy of Sciences, Kazan, Russia; 10 Institute of Biochemistry, Brandenburg Medical School, Neuruppin, Germany

**Keywords:** Cancer metabolism, Cell death

## Abstract

The extent of mitochondrial heterogeneity and the presence of mitochondrial archetypes in cancer remain unknown. Mitochondria play a central role in the metabolic reprogramming that occurs in cancer cells. This process adjusts the activity of metabolic pathways to support growth, proliferation, and survival of cancer cells. Using a panel of colorectal cancer (CRC) cell lines, we revealed extensive differences in their mitochondrial composition, suggesting functional specialisation of these organelles. We differentiated bioenergetic and mitochondrial phenotypes, which point to different strategies used by CRC cells to maintain their sustainability. Moreover, the efficacy of various treatments targeting metabolic pathways was dependent on the respiration and glycolysis levels of cancer cells. Furthermore, we identified metabolites associated with both bioenergetic profiles and cell responses to treatments. The levels of these molecules can be used to predict the therapeutic efficacy of anti-cancer drugs and identify metabolic vulnerabilities of CRC. Our study indicates that the efficacy of CRC therapies is closely linked to mitochondrial status and cellular bioenergetics.

## Introduction

Environmental factors such as nutrient availability, oxygen levels, the degree of inflammation, growth factors and cytokines, as well as the composition of the extracellular matrix, exhibit profound effects on cancer cells [1–3]. These environmental conditions apply selective pressure on tumour cells resulting in the propagation of those cell clones that are best adapted to the unique environment. Such clones may possess specialised types of mitochondria that are instrumental in providing metabolic plasticity and unique adaptations to the existing environmental conditions [4]. Ultimately, these advantageous mitochondrial adaptations support rapid cell growth and proliferation [5–9].

Metabolic reprogramming of cancer cells is tightly linked to mitochondria, owing to the fact that the tricarboxylic acid cycle (TCA cycle) and adjacent pathways are heavily integrated into cellular metabolism [10]. Moreover, mitochondria are not static organelles, rather they can change their morphology, number, and function depending on environmental conditions, such as nutrient deprivation or hypoxia [11–13]. The mitochondrial protein composition can also vary noticeably, indicating a high potential for functional specialization [14–16]. Besides cellular bioenergetics, mitochondria also play a role in other processes crucial for cell maintenance, including redox balance, calcium signalling, lipid and nucleotide synthesis, as well as epigenetic modifications. Alterations and intensifications of these processes are common in cancer cells [12, 17, 18]. Mitochondria-mediated reactive oxygen species (ROS) production is a significant contributor to genetic instability in cancer cells, facilitating the rapid evolution of cancer cell clones. Elevated ROS levels increase the need for ROS scavenging activity, thus presenting a potential vulnerability in cancer cells [19]. Because of these multifaceted roles, targeting the mitochondria and mitochondrial metabolism is considered a promising strategy for cancer therapy [20–22].

Given that metabolism is inherently linked to mitochondria, metabolic alterations could be associated with particular mitochondrial subtypes. Therefore, understanding the overall level of mitochondrial structural and functional heterogeneity in various cancer cells is essential. Currently, it remains unknown whether mitochondrial archetypes exist in cancer.

Appropriate mitochondrial function is highly sensitive to numerous perturbations, including variations in mitochondrial protein ratios, mutations in mitochondrial DNA (mtDNA), issues with mtDNA maintenance, mitophagy, and pharmacological effects. For example, incorrect protein ratios can disrupt the assembly of electron transport chain (ETC) complexes, leading to mitochondrial dysfunction [23, 24]. Similarly, mutations in mtDNA can impair the function of the ETC proteins, affecting the entire mitochondrial structure [24, 25]. Notably, many of these changes are not detectable at the transcriptomic level, occurring instead post-translationally or during mitochondrial import and assembly, which limits the effectiveness of transcriptomic studies in assessing mitochondrial function, particularly in cancer contexts.

There has been a historic misconception that hypoxia almost entirely diminishes oxidative respiration (OXPHOS) levels, leading to a substantial underestimation of the role of mitochondria in cancer. In fact, many cancer cells are still able to obtain a significant portion of their energy through OXPHOS under hypoxia [26–28]. Moreover, recent studies indicate that some cancer cells even rely on OXPHOS for energy production [29–31].

In this study, we aimed to assess the level of mitochondrial heterogeneity and its interaction with bioenergetics, metabolism and drug response in cancer. We focused on colorectal cancer (CRC), anticipating that the high molecular heterogeneity inherent to these cells would yield a diverse range of mitochondrial, bioenergetic and metabolic phenotypes characterizing different CRC subgroups [32–35]. Following the dissection of key mitochondrial features that distinguish CRC cells, we analysed their bioenergetic and metabolic profiles, subsequently classifying them into glycolytic or OXPHOS-predominant subgroups. Based on this divergence, we hypothesized that the effectiveness of therapies targeting metabolic pathways would depend on the mitochondrial state and the bioenergetic phenotype of CRC cells. We pharmacologically inhibited various metabolic pathways and analysed the anti-cancer effects of the utilized compounds. By exploring the link between CRC cell bioenergetics and metabolic profiles, we identified key metabolites that not only correlated with either mitochondrial respiration or glycolysis dependency, but also with drug responses. The presence of these metabolites in particular cancer cells points towards possible metabolic vulnerabilities that can be exploited therapeutically. Furthermore, the levels of these metabolites can serve as biomarkers for predicting the efficacy of anti-cancer treatments.

## Materials & Methods

### Cell culture

CRC cell lines SW480, SW1417, LS123, SW403, SW620, SW48, COLO-320 and HCT-15 were cultured in DMEM medium (Thermo Fisher Scientific, Waltham, Massachusetts, United States; Cat. # 31966047) containing 100 U/ml of penicillin/streptomycin and 10% FBS at 37 °C in an incubator supplied with 5% of CO_2_. Cells were split every 3-4 days using trypsin-EDTA solution (Thermo Fisher Scientific, Cat. # 25200072). All experiments were conducted simultaneously across all cell lines to ensure consistency and relevance. Regular mycoplasma testing was performed, and only low-passage cells were used to maintain experimental integrity. Biological replicates assessing fundamental characteristics such as proliferation rate, mitochondrial biomass, and MMP were performed within a 4–6 week period using the same cell batches. The tight consistency of results across different replicates suggests that these parameters are relatively stable, indicating that cells neither lose nor accumulate mitochondria significantly over several weeks.

### *Analysis of proliferation rate*

CRC cells were stained with 3 μM of the CFSE-like dye CellTrace Violet (Thermo Fisher Scientific, Cat. # C34557) in DPBS solution containing 0.1% FBS for 10 min. Subsequently, stained cells were washed twice with DPBS containing 10% FBS and seeded in standard growth media. The next day the reference control cells were trypsinised, washed, and analysed using a BD FACSLyric™ flow cytometer (BD Biosciences, Franklin Lakes, New Jersey, United States). The purpose of this procedure was to measure the basal level of fluorescence quantified as the geometric mean intensity at the initial time point of the experiment (D_1_). The remaining cells were kept as controls or treated with various compounds and then cultured for 72 h. Subsequently, cells were trypsinised, washed, and analysed by flow cytometry. The cellular proliferation rate was determined by calculating the ratio of the fluorescence signal from the initial reference control sample at D_1_ to the signal obtained at the end of the experiment (D_4_). Proliferative output was calculated by multiplying the cell proliferation rate by cell volume.

### *Expression analysis of mtDNA and nuclear-encoded mitochondrial genes*

The files containing transcriptomic data of untreated CRC cells were obtained from DepMap Portal https://depmap.org/portal/download/all. For analysis, we utilised CCLE_RNAseq_20180929 datasets. The lists of mitochondrial proteins were compiled using Mito Carta 3.0 and MitoProteome (1/18/2022) databases. Subsequently, expression values of 1663 nuclear genes encoding mitochondrial proteins with confirmed mitochondrial localisation and 37 mtDNA-encoded genes were extracted. The expression values were mean normalized, and the median expression level for each cell line was calculated.

### Quantification of mtDNA copy number

mtDNA copy number was quantified and normalized against nuclear DNA copy number using two sets of primers to ensure coherence of the results. Initially, cellular DNA (both nuclear and mitochondrial) was extracted using proteinase K treatment, isopropanol DNA precipitation and ethanol purification. Subsequently, 50 ng of DNA was amplified using qPCR and two sets of primers, either ATP8/B2M: ATP8 (mtDNA) forward 5′-AATATTAAACACAAACTACCACCTACC-3′ and reverse 5′-TGGTTCTCAGGGTTTGTTATA-3′; B2M (gDNA) forward 5′-TGCTGTCTCCATGTTTGATGTATCT-3′ and reverse 5′-TCTCTGCTCCCCACCTCTAAGT-3′; or tRNALeu/LDL: tRNALeu (mtDNA) forward 5′-CACCCAAGAACAGGGTTTGT-3′ and reverse 5′-TGGCCATGGGTATGTTGTTA-3′; LDL (gDNA) forward 5′-CGAGTCGTCTTTCTCCTGATGAT-3′ and reverse 5′-TTCTGGATTCCAATGCTTCGA-3′. mtDNA copy number was calculated using the 2^ΔCt^ formula, where ΔCt = gDNA Ct – mtDNA Ct.

### Measurement of mitochondrial membrane potential, quantification of cells with depolarized mitochondria and evaluation of mitochondrial ROS levels

CRC cells were trypsinized and resuspended in complete media containing 3 μM of JC-1 dye. Cells were incubated for 30 min at 37 °C and 5% CO_2_. Afterwards, cells were acquired with a BD FACSLyric™ flow cytometer (BD Biosciences). The red/green ratio characterising the level of MMP was calculated by dividing the geometric mean fluorescence intensity detected by the PE channel (red) by the geometric mean fluorescence intensity detected by the FITC channel (green). Additionally, cells were stratified by their ability to form J-aggregates characterising the presence of mitochondria with a high MMP. The signal from J-aggregates was detected in the PE (red) channel. Cells with a low signal in this channel were identified as cells with low MMP, which were primarily characterised by depolarized mitochondria.

For mitochondrial ROS evaluation, CRC cells were resuspended in serum-free media containing 0.1% BSA and 5 μM of MitoSox Red dye (Thermo Fisher Scientific, Cat. # M36008). Cells were incubated for 30 min at 37 °C and 5% CO_2_. Afterwards, cells were acquired with a BD FACSLyric™ flow cytometer (BD Biosciences).

### *Western blotting*

Protein samples were prepared by lysing CRC cells in RIPA buffer with a protease inhibitor cocktail (Sigma-Aldrich, St. Louis, Missouri, United States; Cat. # P8340) and 1 mM PMSF. 30 μg of extracted proteins were denatured with 100 mM dithiothreitol and LDS Sample Buffer (Abcam, Cambridge, United Kingdom; Cat. # ab119196). Samples were subjected to electrophoretic separation followed by protein transfer onto PVDF membrane (Merck Millipore, St. Louis, Missouri, United States; Cat. # IPVH00010). Membranes were blocked in 5% non-fat milk in TBST for 1 h and incubated with primary antibodies (Total OXPHOS Human WB Antibody Cocktail, Abcam, Cat. # ab110411; TOMM20, Abcam, Cat. # ab186735; beta-actin, GenScript, Piscataway, New Jersey, United States; Cat. # A00730) at 4 °C overnight. Subsequently, the membranes were washed with TBST and incubated with the corresponding HRP-conjugated secondary antibody (GE Healthcare, Chicago, Illinois, United States). The proteins were detected by the induction of chemiluminescence using the Clarity Western ECL Substrate (Bio-Rad, Hercules, California, United States; Cat. # 1705061) by the ChemiDoc XRS+ system (Bio-Rad).

### Isolation of mitochondria and mass spectrometry

Mitochondrial samples originating from three independent biological replicates were isolated from corresponding CRC cell lines using Mitochondria Isolation Kit for Cultured Cells (Abcam, Cat. # ab110170) and kept frozen at −80 °C until further analysis. Prior to the analysis, mitochondrial samples were treated with benzonase and SDS, then lysed via sonication and centrifuged to extract the supernatant. Proteins were precipitated using the chloroform-methanol method, denatured in a urea-thiourea buffer, and protein concentrations were measured by the Bradford assay. Disulfide bonds in the proteins were reduced and alkylated before being digested with trypsin/Lys-C. The digested peptides were desalted, dried, and prepared for LC-MS/MS analysis. In the LC-MS analysis, peptides were separated on a capillary column and eluted over 60-min using 0.1% formic acid in an acetonitrile mobile phase. The analysis was conducted on a high-resolution quadrupole-orbitrap tandem mass-spectrometer Exploris 480 (Thermo Fisher Scientific). The electrospray ion source was set at 2200 volts, scanning ions from m/z 200 to 1500. Precursors were fragmented using fixed collision energy, and the resulting peptides were detected and analysed.

### *Analysis of proteomics data*

For the analysis of proteomics data, MaxQuant software version 2.4.2.0 was used. The analysis included carbamidomethyl as a constant modification and both deamidation and oxidation as variable modifications. Peptide parameters were set to lengths of 8–25 amino acids with a maximum mass of 4600 Da. Initially, we detected peptides corresponding to approximately 800 different mitochondrial proteins, however after the calculation of LFQ values and EigenMS normalization only 453 proteins passed quality control thresholds. The lists of mitochondrial proteins were compiled using Mito Carta 3.0 and MitoProteome (1/18/2022) databases. Differentially expressed proteins (DEPs) were identified by Limma using 0.1 as FDR and 2 as fold-change cut-offs. The DEPs obtained from all comparisons were then used for enrichment analysis using IDEP 2.0 tool and various enrichment libraries such as GO Biological Process, GO Molecular Function, GO Cellular Component, KEGG, Reactome, WikiPathways, Hallmark MSigDB, Disease Alliance, curated RGD, Elsevier, and GeneSetDB. For each enrichment term in every comparison, we extracted the fold enrichment values and calculated their sums relative to the other cell lines. This approach enabled us to derive fold enrichment scores for each cell line, indicating whether the proteins within each enrichment term were predominantly upregulated, downregulated, or unchanged compared to the other seven cell lines. Subsequently, we performed a Z-transformation of the obtained values to standardize them for further analysis. To address the redundancy among the 2248 enrichment terms sourced from 11 different libraries, we grouped similar terms. These terms, often named similarly, reflect the same processes, but do not contain exactly the same sets of genes. Therefore, the results of enrichment analysis were highly depended on the usage of a particular enrichment library. In order to address this bias, we created “consensus enrichment scores”, which represent the average of *Z*-scores belonging to similar terms. This approach clarifies whether an enrichment term is upregulated/downregulated in multiple different enrichment libraries and therefore excludes the database-dependent bias. As alternative to pairwise DEPs-based enrichment analysis, we also utilized PGSEA-based enrichments analysis on all proteins using IDEP 0.96 and the built-in PGSEA tool.

### *Measurement of mitochondrial biomass*

CRC cells were trypsinised, washed, and resuspended in complete media containing either 200 nM of MitoTracker™ Green FM (Thermo Fisher Scientific, Cat. # M7514) or 100 nM of MitoTracker™ Deep Red FM (Thermo Fisher Scientific, Cat. # M22426) for 30 min at 37 °C and then acquired with a BD FACSLyric™ flow cytometer (BD Biosciences).

### Analysis of cellular bioenergetic profiles using Seahorse XFe96

To minimize the interference of external factors, the metabolic profiles of all cell lines were measured simultaneously. The number of seeded cells in the XFe96 cell culture microplate varied in the range of 1.5 × 10^4^–1 × 10^5^ owing to their difference in size. The final results were normalized using the initial seeding coefficients. In order to avoid additional variability induced by an uneven proliferation rate of examined cell lines, the cells were seeded on poly-L-lysine coated plates allowing for rapid adhesion and immediate execution of the assay. The assay was executed in XF DMEM Medium pH 7.4 media (Agilent, Santa Clara, California, United States; Cat. # 103575-100) containing 10 mM glucose (Agilent, Cat. # 103577-100), 2 mM L-glutamine (Agilent, Cat. # 103579-100) and 1 mM sodium pyruvate (Agilent, Cat. # 103578-100). Next, cells were incubated at 37 °C in a non-CO2 incubator for 1 h. Oxygen consumption rate (OCR) and extracellular acidification rate (ECAR) were measured under basal conditions and in response to sequentially injected compounds at a final concentration of 1.5 μM oligomycin (Sigma-Aldrich Cat. # O4876), 2 μM FCCP (Sigma-Aldrich Cat. # C2920), and 1.5 μM rotenone (Sigma-Aldrich Cat. # R8875) + 1.5 μM antimycin A (Sigma-Aldrich Cat. # A8674) and 50 mM of 2-deoxy-D-glucose (Sigma-Aldrich Cat. # D6134). Stocks of compounds were prepared in the same DMEM media and loaded into delivery ports of Seahorse cartridges. The design of the assay allowed calculation of multiple parameters related to glycolysis and mitochondrial respiration in examined cells. The parameters related to respiration were assessed, including non-mitochondrial respiration (OCR after rotenone+antimycin A injection), basal mitochondrial respiration (ΔOCR between the steady state and after rotenone+antimycin A injection), ATP-linked respiration (ΔOCR between basal mitochondrial respiration and after oligomycin injection), maximal mitochondrial respiration (ΔOCR between non-mitochondrial respiration and after FCCP injection). The parameters related to glycolysis were assessed, including non-glycolytic acidification (ECAR after 2-DG injection), glycolysis (ΔECAR between the steady state and after 2-DG injection), maximal glycolytic capacity (ΔECAR between non-glycolytic acidification and after oligomycin injection), glycolytic reserve (ΔECAR between the steady state and after oligomycin injection).

### Quantification of cell numbers

The quantification of cell numbers at the final stage of the experiment was conducted using the BD FACSLyric™ flow cytometer (BD Biosciences), capitalizing on the capability of the flow cytometer to aspirate and count cells at a fixed speed. In brief, the supernatant containing both floating and dead cells was harvested. Subsequently, the obtained supernatant corresponding to each particular sample was added to inactivate trypsin and for cell resuspension thereby minimizing cell loss. The harvested cells were neither washed nor centrifuged in order to prevent any potential cell loss. A portion of each sample was subjected to vortexing, followed by immediate measurement for a duration of 45 s.

The total number of cells present in the sample was then calculated based on the obtained count from the measured sample fraction. Only events corresponding to cells characterized by normal size were considered.

### Assessment of apoptosis levels

Apoptosis levels in CRC cells were identified using annexin V assay. Briefly, trypsinized cells were washed and resuspended in 50 μl Annexin V binding buffer pH 7.4 containing 10 mM Hepes, 140 mM NaCl and 2.5 mM CaCl_2_. Subsequently, cells were incubated with 1.25 μl of APC Annexin V probe (BD Biosciences Cat. # 550475) for 15 min at room temperature. After incubation, the cells were mixed with an additional 100 μl of Annexin V binding buffer and analysed using a flow cytometer BD FACSLyric™ flow cytometer (BD Biosciences).

### *Consensus enrichment score*

For the enrichment analysis of DEPs isolated from the mitochondria of 8 CRC cell lines, we utilized 11 different enrichment libraries (GO terms, KEGG, Reactome, WikiPathways and others) in order to avoid bias linked to the selection of one particular library. The resulting analysis led to the enrichment of 2248 unique enrichment terms. Subsequently, we observed significant overlap in terminology across different databases, often representing functionally similar processes. For example, for terms like “valine, leucine, and isoleucine degradation” we found 3 matches:

**Table:** 

KEGG	Valine leucine and isoleucine degradation
GeneSetDB	Valine leucine and isoleucine degradation
RGD	Valine leucine and isoleucine degradation pathway

Although the protein lists associated with these terms showed a high degree of overlap, they were not identical, indicating that relying on a single library could potentially skew results and conclusions. To mitigate this bias and avoid cherry-picking results, we developed a consensus enrichment score. We first converted fold enrichment values from all libraries to *Z*-scores. Subsequently, terms representing similar biological processes, like the above-mentioned “valine, leucine, and isoleucine degradation” examples, were grouped together. Finally, the average *Z*-score within each group was calculated and designated as the “consensus enrichment score.” This score reflects the enrichment strength for a particular biological process, integrating information from multiple databases.

### *Drug response correlation score*

To better understand the relationship between mitochondrial/bioenergetic characteristics and cell responses to drug treatments, we introduced a “drug response correlation score.” This score integrates the treatment-induced changes in cell number, proliferation rate, and apoptosis levels, with weighted values to reflect their significance (see Fig. S15A). Specifically, the change in cell number is given a weight of 1.0, acknowledging its primary importance, while changes in proliferation and apoptosis are each weighted at 0.5. Thus, the score is calculated as the sum of these weighted correlation coefficients, represented as 1.0 × *P*_*CCvsBM*_ (treatment-induced change in cell number vs basal metric) + 0.5 × *P*_*PCvsBM*_ (treatment-induced change in proliferation rate vs basal metric) + 0.5 × *P*_*ACvsBM*_ (treatment-induced change in apoptosis levels vs basal metric), where *P* denotes Pearson correlation coefficients. This method provides a more precise assessment of drug effects, ranging from −2 to 2, thereby offering a broader range than the conventional Pearson coefficient scale of −1 to 1.

### Bioenergetic score

Considering that abundance of any metabolite can be simultaneously linked with both mitochondrial respiration and glycolysis levels, we aimed to utilize this dual association to enhance the resolution of our analysis. To achieve this, we introduced a “bioenergetic score” that integrates a metabolite’s correlation with glycolysis and mitochondrial respiration into a single value (see Fig. S15C). This bioenergetic score is calculated by subtracting the Pearson correlation coefficient for glycolysis from the Pearson correlation coefficient for mitochondrial respiration. A higher score indicates that a metabolite is positively associated with OXPHOS and negatively with glycolysis, whereas a lower score suggests that a metabolite is negatively associated with OXPHOS and positively with glycolysis. This scoring system allows for a broader assessment of metabolite association with bioenergetic processes, providing a range from −2 to 2, compared to the conventional Pearson coefficient range of −1 to 1.

### *Data retrieval & statistical analysis & software*

**Fig. 1:**
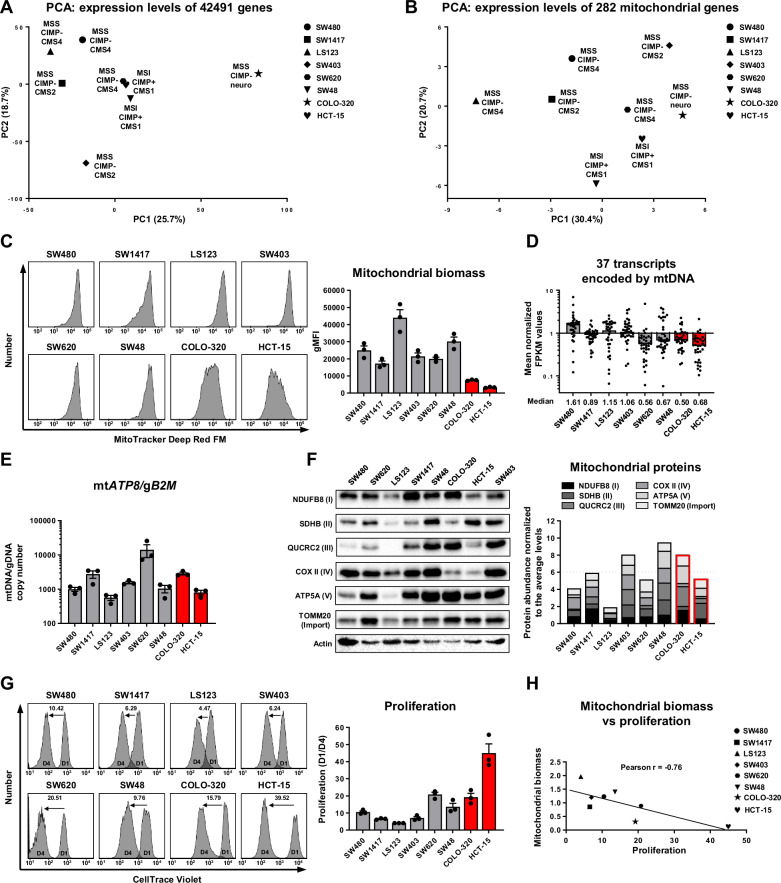
Multi-parameter analysis of mitochondrial biomass in CRC cell lines. **A** PCA of transcriptomic data extracted from the Broad DepMap Portal and CCLE project visualising the RNA FPKM values expressed in the examined cell lines. **B** PCA of transcriptomic data visualising the RNA FPKM values of genes critical for mitochondrial function. **C** Representative histograms and their quantification depicting mitochondrial biomass assessed by flow cytometry analysis of CRC cells stained with 100 nM MitoTracker Deep Red FM dye. Values are means ± SEM, *n* = 3. **D** Mean normalized FPKM values of transcripts encoded by mtDNA, with each dot representing one gene. Data extracted from the CCLE project. **E** qPCR-quantified mtDNA copy number normalized against genomic copy number. Values are means ± SEM, *n* = 3. **F** Western blot analysis of 6 mitochondrial proteins and actin in lysates of CRC cells and the quantification of protein abundance are represented as stacked bar charts illustrating their combined levels. **G** Representative histograms and quantification of flow cytometry analysis characterising the proliferation rate of CRC cells. The cells were stained with 3 μM of CellTrace Violet dye and analysed after 24 h (D1) and 96 h (D4), proliferation rate was calculated as the D1/D4 ratio. Values are means ± SEM, *n* = 3. **H** Pearson correlation reflecting the association between mitochondrial biomass (average of mean normalized MitoTracker Green and Deep Red signals) and proliferation rate in CRC cells.

The files containing transcriptomic and metabolomic data of untreated CRC cells were obtained from DepMap Portal https://depmap.org/portal/download/all. Particularly for this project, we utilised CCLE_metabolomics_20190502 and CCLE_RNAseq_20180929 datasets. The compilation of 338 genes encoding critical mitochondrial proteins, particularly those integral to mitochondrial function, was derived from the work “*Genetics of mitochondrial diseases: Identifying mutations to help diagnosis”* by Stenton & Sarah L, 2020. For the PCA in Fig. 1C 282/338 of these genes were used. The PCA of transcriptomic, bioenergetic and metabolomic data was done using SVD with imputation method in ClustVis online tool https://biit.cs.ut.ee/clustvis/. The PCA and the PCA loadings of the drug responses were created using the PCA function with the selected standardize option in GraphPad Prism 9.5.1 software. Visualisation of data was done using GraphPad Prism 9.5.1 and FlowJo 10.9 software, with the latter also used for the analysis of flow cytometry data. Hierarchical and K-means clustering of metabolic data in CRC cells was performed using the one minus Pearson correlation metric in the Morpheus tool https://software.broadinstitute.org/morpheus/. All measurements were conducted on distinct samples representing biological replicates, with the exception of Fig. 3C, D. These figures are representative, featuring technical replicates to illustrate the results of a single experiment.

**List of drugs and compounds**

**Table:** 

Compound:	Primary target:	Concentration:	Source:	Catalogue number:
Rotenone	Complex I	0.2, 1.5 μM	Sigma-Aldrich	Cat. # R8875
Antimycin A	Complex III	0.2, 1.5 μM	Sigma-Aldrich	Cat. # A8674
Oligomycin	Complex V	0.1 μM	Sigma-Aldrich	Cat. # O4876
CCCP	Mitochondrial uncoupler	50 nM	Sigma-Aldrich	Cat. # 21855
FCCP	Mitochondrial uncoupler	2 μM	Sigma-Aldrich	Cat. # C2920
UK5099	Pyruvate	20 μM	Sigma-Aldrich	Cat. # PZ0160
Etomoxir	Fatty acid oxidation	5 μM	Sigma-Aldrich	Cat. # E1905
BPTES	Glutaminolysis	0.5 μM	Sigma-Aldrich	Cat. # SML0601
Dimethyl fumarate	TCA cycle	10 μM	Sigma-Aldrich	Cat. # 242926
Mdivi-1	Fusion/fission dynamics	20 μM	Enzo Life Sciences	Cat. # CM1270010
Cyclosporine A (CsA)	MPTP	200 nM	Sigma-Aldrich	Cat. # C1832
2-Deoxy-D-Glucose (2-DG)	Glycolysis	200 μM	Sigma-Aldrich	Cat. # D6134
AZD3965	Inhibits monocarboxylate transporters	10 nM	MedChemExpress	Cat. # HY-12750
EIPA (Ethylisopropylamiloride)	Inhibits sodium-hydrogen exchangers	1 μM	MedChemExpress	Cat. # HY-101840
Topiramate	Inhibits carbonic anhydrases	1 μM	MedChemExpress	Cat. # HY-B0122
Concanamycin A	Glycolysis	2 nM	Santa Cruz Biotechnology	Cat. # sc-202111
Irinotecan	Blocks topoisomerase-1	2 μM	MedChemExpress	Cat. # HY- 16562
Oxaliplatin	Crosslinks DNA	2 μM	MedChemExpress	Cat. # HY-17371
5-Fluorouracil (5-FU)	Inhibitor of thymidylate synthase	5 μM	Sigma-Aldrich	Cat. # F6627

## Results

### A subgroup of CRC cells displays drastically reduced mitochondrial biomass

CRC exhibit remarkable heterogeneity dividing them into four molecular subtypes (CMS1-4), which are additionally characterized by features such as microsatellite instability or the CpG island methylator phenotype [33, 34]. CRC cells also frequently harbour mutations in the TP53, KRAS, BRAF, and PTEN genes. In line with their high metastatic potential, CRC cell lines originate from diverse tissues and various locations, including different segments of the large intestine, lymph nodes, or metastatic sites. This diversity is further heightened by variable oxygen levels across different colon regions [36, 37]. Considering this variability, we presumed that CRC cells could exhibit significant differences in their mitochondrial function [38–40].

To test this hypothesis, we assembled a panel of eight highly diverse CRC cell lines belonging to different CMS subtypes and driven by different mutations, thereby excluding the bias associated with the selection of a particular CRC subtype in our study (Fig. S1A). Initially, we analysed a publicly available RNA-seq dataset from the Broad DepMap Portal, which provides expression profiles of CRC cells under steady-state conditions and conducted principal component analysis (PCA) on all expressed genes and a subset of genes critical for mitochondrial function extracted from Stenton et al. (Fig. 1A, B) [41]. Initial PCA findings showed that COLO-320 cells, which are of the a neuroendocrine origin, markedly diverged from other lines. The analysis revealed a significant variability and the presence of numerous differentially expressed genes, with cell lines sharing the same CMS status exhibiting clustering tendencies. A PCA focussing on 282 mitochondrial protein-encoding transcripts showed distinct expression patterns without apparent clustering according to cancer subtype, suggesting variable mitochondrial function across the cell lines.

To assess differences in the mitochondrial function between the examined cells, we evaluated the levels of mitochondrial biomass using two MitoTracker stainings (Figs. 1C and S1B). Both stainings demonstrated similar results, with the highest amount of mitochondria in LS123 and drastically reduced levels in the COLO-320 and HCT-15 cell lines. Such a high extent of variability prompted us to examine mtDNA transcripts levels, which generally reflect the mitochondrial abundance. Analysis of all 37 mtDNA-encoded genes revealed diminished transcript levels in SW620, SW48, COLO-320, and HCT-15, suggesting either a reduced amount of mitochondria in these cells or potential impairment of mtDNA maintenance (Fig. 1D). To assess the mtDNA status, we measured mtDNA copy numbers, normalized to genomic DNA (Figs. 1E and S1C). All cell lines showed high mtDNA copy numbers, indicating functional mtDNA maintenance machinery. However, the nuclear-encoded mitochondrial gene expression was not uniformly reduced; it was lowest in LS123, despite their high mitochondrial biomass (Fig. S1D). This discrepancy prompted us to investigate the mitochondrial protein density using Western blot analysis, which revealed the lowest protein density in LS123 (Fig. 1F). Considering the lack of correlation between the total mitochondrial biomass and the protein density, we explored additional factors that might be critical during the mitochondrial assessment in CRC cells. We found that cell volume was another significant variable. Using the forward scatter width and area as proxies for cell diameter and assuming spherical cell geometry, we calculated relative cell volumes and mitochondrial densities (Fig. S1E–G). LS123 cells, being on average larger than other cell lines, had higher mitochondrial content. However, upon normalisation for the cell volume, SW403 and SW48 showed the highest mitochondrial densities, whereas COLO-320 and HCT-15 consistently displayed the lowest values. Collectively, these findings suggest that impairment of mitochondrial assembly or turnover, rather than nuclear gene expression, predominantly accounts for reduced mitochondrial biomasses in COLO-320 and HCT-15.

Given that mitochondria play the primary role in the process of energy production, we correlated the mitochondrial abundance with CRC cell proliferation rates (Fig. 1G, H). COLO-320 and HCT-15 exhibited notably high proliferation rates, linking the mitochondrial loss to the increased proliferative capacity and suggesting that biomass optimisation is an essential cancer adaptation in certain CRC subgroups.

### Mitochondria of CRC cells display high heterogeneity and functional specialization

**Fig. 2:**
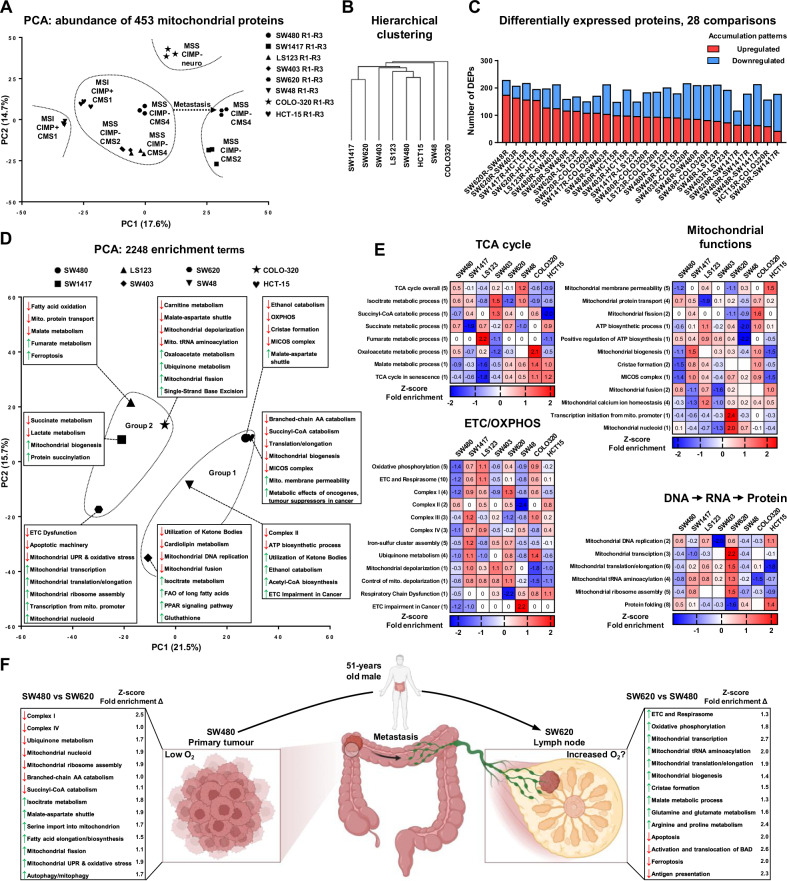
Proteomics and functional enrichment analysis of CRC mitochondria. **A–F** Label-free LC-MS/MS proteomic analysis of mitochondria extracted from three biological replicates across 8 CRC cell lines. **A** PCA of proteomic data characterizing the abundance of 453 mitochondrial proteins in extracted mitochondria. **B** Hierarchical clustering of mitochondria derived from CRC cell lines based on the abundance of 453 mitochondrial proteins. **C** Depiction of the number of upregulated and downregulated differentially expressed proteins derived from 28 pairwise comparisons between mitochondria isolated from eight CRC cell lines. **D** PCA of Z-transformed fold enrichment values corresponding to enrichment of 2248 terms from 11 libraries. **E** Heatmaps illustrating average *Z*-score values. Values reflect the average of multiple Z-transformed fold enrichment scores. The number in brackets indicates the number of similar enrichment terms used for the creation of “the consensus enrichment term”. Number 1 reflects that only one original term was used. **F** Comparison between parent SW480 and SW620 cell lines derived from primary and metastatic sites, respectively. Numbers indicate the *Z*-score delta of fold enrichment scores between these two cell lines.

Intrigued by the substantial variability in mitochondrial biomass observed among CRC cell lines, we hypothesized that different CRC subgroups may favour specific mitochondrial functions or exhibit unique mitochondrial adaptations. To explore this, we isolated mitochondria from corresponding cell lines and performed proteomics analysis. We obtained values corresponding to the abundance of 453 mitochondrial proteins enabling direct comparisons between examined CRC cell lines. Initial PCA and hierarchical clustering revealed a high level of heterogeneity among the mitochondria from different CRC cell lines (Fig. 2A, B). While PCA suggested a slight tendency towards clustering by CMS, hierarchical clustering identified four distinct mitochondrial subgroups, not aligned with CMS classification: (1) COLO-320; (2) SW48; (3) SW1417 and SW620; (4) SW403, LS123, SW480, and HCT-15.

To identify the primary differences among investigated mitochondria, we utilized K-means clustering on the 273 most variable proteins (Fig. S2A). Subsequently, we visualized the distribution of these proteins across eight clusters and conducted a STRING-based interaction analysis and an enrichment analysis for each cluster (Fig. S2B, C). The analysis revealed significant networks of downregulated and upregulated proteins that functionally distinguish the mitochondria of different CRC cell lines, particularly evident in clusters 4 and 7. These clusters highlighted the major downregulation of various interconnected proteins in the SW403 and SW48 cell lines, respectively, suggesting the presence of potential cell line-specific mitochondrial dysfunction.

Furthermore, in clusters 1, 5, and 7, we observed the enrichment of terms closely associated with CRC metabolic reprogramming, including “GUCYC2C signaling in CRC,” “WP4290 Metabolic reprogramming in CRC,” and “Metabolic Reprogramming in Cancer.” These findings imply that a significant fraction of the metabolic adaptations in CRC cells occur in or are driven by mitochondrial changes, underlining the critical role of mitochondrial rearrangements in the metabolic reprogramming of CRC.

We next evaluated pairwise differences and quantified the DEPs between all cell lines, resulting in 28 pairwise comparisons (Fig. 2C). The DEPs were used for enrichment analysis, integrating and Z-transforming fold enrichment values from various databases to create “consensus enrichment scores” for further analysis (see Materials & Methods). This analysis identified 2248 unique terms, underscoring substantial structural and functional differences between the mitochondria (Fig. S3A). PCA and hierarchical clustering based on the enrichment scores of these terms suggested that CRC mitochondria could potentially be divided into two groups: (1) SW480, HCT-15, SW48, and SW403, and (2) SW1417, SW620, COLO-320, and LS123 (Fig. 2D). The primary features derived from consensus enrichment score analysis and PGSEA-based enrichment analysis are emphasized in the PCA and following heatmaps (Figs. 2E and S3B, C). In brief, we observed that mitochondrial composition varies drastically in terms of the expression of different enzymes involved in the catabolism of various mitochondrial fuels. This includes enzymes responsible for fatty acid oxidation (FAO), catabolism of branched-chain amino acids, glutaminolysis, utilization of ketone bodies, pyruvate metabolism, and acetyl-CoA biosynthesis. These variations suggest that some cancer cells may adapt their mitochondria based on the availability of substrates, allowing them to utilize a broad range of metabolic substrates to diversify their energy sources. Moreover, we noted significant differences in the expression of TCA cycle enzymes, ETC/OXPHOS machinery, and proteins essential for maintaining core mitochondrial functions among the examined mitochondria. These data suggest that the mitochondria of CRC cells might evolve and adapt in conjunction with the cancer cells themselves.

Finally, we compared the SW480 and SW620 cell lines, derived from the same patient but representing primary and metastatic sites, respectively (Fig. 2F). Surprisingly, key mitochondrial functions like the activity of ETC complexes, OXPHOS, mtDNA replication/transcription, and mitochondrial translation were predominantly upregulated in the metastatic SW620 cell line. This may be due to the higher oxygen concentrations in lymph nodes compared to the hypoxic primary tumor environment, allowing metastatic cells to enhance their OXPHOS machinery. Additionally, this cell line downregulated proteins linked to apoptosis/ferroptosis and antigen presentation, indicating unique mitochondrial adaptations that may help these cancer cells avoid cell death and evade immune detection in the metastatic niches.

### CRC cells develop different bioenergetic strategies and display variable mitochondrial functionality

**Fig. 3:**
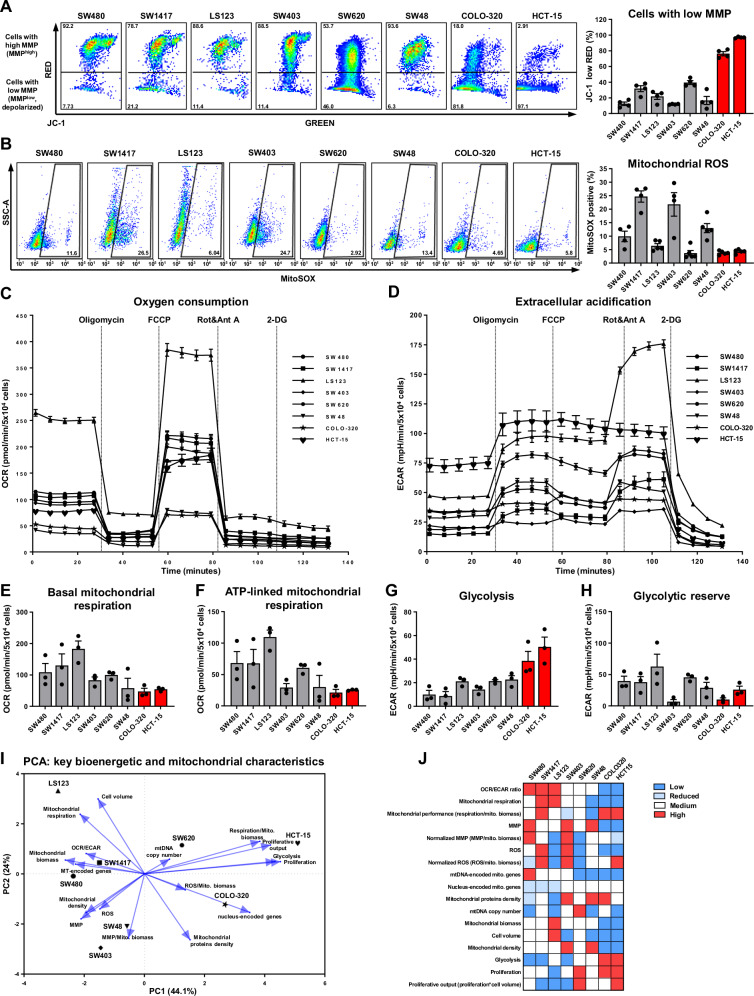
Functional mitochondrial characterization and bioenergetic profiling of CRC cell lines. **A** Representative histograms and quantification, which demonstrate the frequencies of CRC cells with low MMP. The analysis was performed by flow cytometry using 3 μM JC-1 dye. Values are means ± SEM, *n* = 3–4. **B** Representative histograms and quantification of mitochondrial ROS levels. The analysis was performed by flow cytometry using 5 μM MitoSOX Red dye. Values are means ± SEM, *n* = 4–5. **C–H** Bioenergetic analysis of CRC cells using a Seahorse analyser upon subsequent injections of oligomycin, FCCP, rotenone+antimycin A, 2-DG. **C**, **E**, **F** Parameters related to oxygen consumption represented by OCR values. **D**, **G**, **H** Parameters related to extracellular acidification represented by ECAR values. **C**, **D** Representative graphs derived from one experiment. **E**–**H** Quantifications of mitochondrial respiration and glycolysis parameters derived from three independent experiments. Values are means ± SEM, *n* = 3. **I** PCA of 17 parameters characterizing mitochondrial, bioenergetic and cellular features in CRC cells. **J** Heatmap depicting the manually scored distribution of 17 parameters characterizing mitochondrial, bioenergetic and cellular features of CRC cells.

Next, we decided to explore how variations in the mitochondrial composition and biomass affect the mitochondrial performance and bioenergetic profiles in CRC cells. Using JC-1 staining, we found that SW480, SW403, and SW48 cells exhibited a significantly higher mitochondrial membrane potential (MMP) compared to the others (Fig. S4A), correlating with their higher mitochondrial biomass. These cell lines also showed the lowest proportion of cells with low MMP (Fig. 3A). Conversely, COLO-320 and HCT-15 had a high percentage of cells with low MMP (80% to 98%). Furthermore, mitochondrial ROS production, a by-product of the ETC activity (Fig. 3B), was higher in the cell lines with elevated mitochondrial biomass including SW1417, SW403, and SW48. Elevated ROS production in cancer cells can arise from two main scenarios: either cells show high OXPHOS activity or have partially dysfunctional and leaky ETC, leading to enhanced ROS production. Consequently, our findings suggest that certain CRC cells may exhibit mitochondrial dysfunction.

To evaluate how mitochondrial discrepancies affect OXPHOS in CRC cells, we conducted a bioenergetic profiling using the Seahorse XFe96 analyser (Figs. 3C, E, F and S4B, C). The results indicated that COLO-320, HCT-15, SW48, and SW403 cells displayed low levels of respiration-related parameters, particularly the ATP-dependent respiration. While the low respiratory activity in COLO-320 and HCT-15 was anticipated due to their significantly reduced mitochondrial biomass, the same phenomenon was unexpectedly observed in SW48 and SW403 cells, despite their high mitochondrial density. These cells also displayed elevated ROS production and significant downregulation of major mitochondrial proteins, suggesting that they have dysfunctional mitochondria with diminished activity.

In contrast, the cell lines SW480, SW1417, LS123, and SW620 exhibited relatively high levels of respiration. Notably, these cells displayed a significant discrepancy between the absolute mitochondrial biomass and mitochondrial density due to large differences in their cell volumes. To assess their mitochondrial performance, accounting for differences in mitochondrial biomass, we normalized the values of mitochondrial respiration to the number of mitochondria (Fig. S4F). This analysis revealed that while SW48 and SW403 had the lowest normalised mitochondrial performance, COLO-320 and HCT-15 showed the highest, despite the lower absolute respiration rates. This indicates that some CRC cells may have fewer mitochondria, but they are highly active, dealing with substantial cellular OXPHOS demands and promoting high proliferation rates.

On the other hand, despite having the highest mitochondrial biomass and cell volume, LS123 cells showed only average normalized mitochondrial performance, indicating that evaluations based solely on the mitochondrial biomass or respiration can be misleading. Additionally, our analysis highlighted SW1417 cells as having a balanced OXPHOS-predominant phenotype. These cells, with only a moderate mitochondrial count, exhibited high absolute mitochondrial respiration and ROS levels, alongside the robust normalized performance, without significant disruptions in the mitochondrial protein composition.

Complimentary to the mitochondrial respiration profiling, we assessed glycolysis-related parameters in CRC cells (Figs. 3D, G, H and S4D, E). Interestingly, cells characterized by decreased absolute respiration simultaneously exhibited increased glycolytic flux, which was especially pronounced in HCT-15 and COLO-320, and less evident in SW480, SW1417, and SW403 cells. These results demonstrate that cells with reduced OXPHOS are prone to upregulate glycolysis.

An intriguing pattern of “bioenergetic optimization” was observed in the metastatic SW620 cell line, which, like the primary SW480 cell line, is derived from the same patient. The metastatic SW620 showed a slight reduction in mitochondrial biomass but demonstrated increased normalized mitochondrial performance compared to its primary counterpart. Proteomic analysis revealed improved ETC/OXPHOS machinery in SW620 cells, suggesting that despite fewer mitochondria, these mitochondria are more active. This enhanced activity was accompanied by a twofold increase in glycolytic flux, which led to a doubled proliferation rate compared to SW480 cells. Collectively, these findings suggest the existence of an adaptive strategy that enhances metastasis and colonization of new niches.

A summary of these key differences in CRC cells is presented through PCA (Fig. 3I) and a heatmap, which scores the examined mitochondrial and bioenergetic characteristics, highlights the principal differences among the cell lines (Fig. 3J).

### Bioenergetic profiles and metabolite distribution are strongly interconnected in CRC cells

**Fig. 4:**
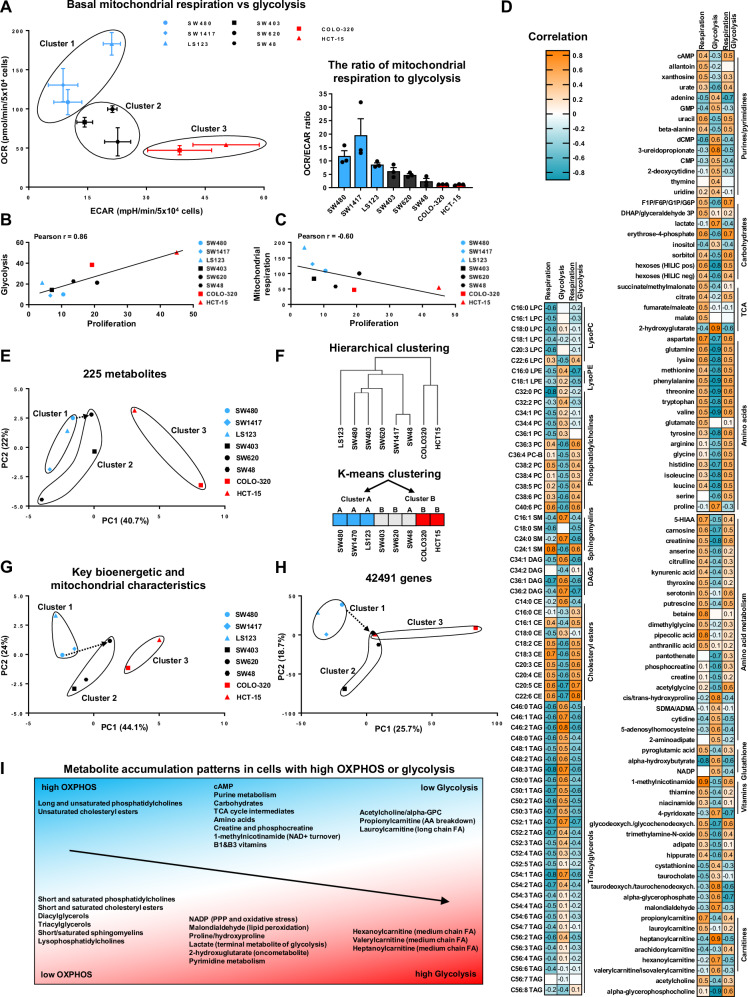
Interconnectivity of bioenergetic phenotypes and metabolite profiles in CRC. **A** Visualisation of OCR and ECAR values derived from bioenergetic analysis and OCR/ECAR ratio representing the relation between basal mitochondrial respiration and glycolysis. Values are means ± SEM, *n* = 3. Bioenergetic clusters are colour-coded by metabolic profile (blue: high OXPHOS, black: intermediate, red: high glycolysis). **B**, **C** Pearson correlations characterising the relations between the proliferation rate of CRC cells and their bioenergetic parameters such as glycolysis and basal mitochondrial respiration, respectively. **D** Correlation analysis linking levels of metabolites and bioenergetics in CRC cells. The heatmaps depict metabolites exhibiting moderate or strong correlations (<−0.45 or > 0.45) between the abundance of metabolites and the rates of glycolysis/mitochondrial respiration/ratio of mitochondrial respiration and glycolysis in CRC cells. **E** PCA of metabolomic data characterizing the abundance of all analysed metabolites in examined cell lines. **F** Hierarchical and K-means clustering of selected CRC cell lines based on the abundance of 225 metabolites. **G**, **H** PCAs illustrating a high level of interconnection between bioenergetics and mitochondrial characteristics as well as the CRC cell transcriptome. Similar plots depicting PCAs are shown in Figs. 1A and 3I and represented here again as a summary. The arrows illustrate the transition in the status of closely related SW480 and SW620 cell lines derived from the same patient and collected from primary and metastatic sites, respectively. **I** Schematic representation of the main patterns of metabolite accumulation either in glycolytic cells or in cells relying on mitochondrial respiration.

To define the main bioenergetic classes characterizing CRC cells, we summarized the results of the Seahorse analysis as OCR/ECAR ratios illustrating metabolic dependencies and plotted corresponding values to evaluate the separation between the examined cell lines (Fig. 4A). The obtained results defined three bioenergetic groups: a high OXPHOS, low glycolysis group (cluster 1, blue); an intermediate group with reduced respiration or elevated glycolysis (cluster 2, black); and a predominantly glycolytic group with minimal mitochondrial respiration (cluster 3, red). Additionally, while exploring the relationship between bioenergetics and cell proliferation, we found strong but contrasting correlations. Specifically, glycolysis exhibited a positive correlation with proliferation, whereas mitochondrial respiration showed a negative correlation, linking a lower OCR/ECAR ratio with higher proliferation rates (Fig. 4B, C).

Next, we aimed to understand how bioenergetics correlate with the metabolome of CRC cells. To this end, we analysed a dataset from the DepMap Portal detailing the steady-state abundance of 225 metabolites in CRC cells (Fig. S5A–J). We focused on metabolites displaying at least moderate correlations with the rates of mitochondrial respiration, glycolysis, or their ratio (Fig. 4D). Furthermore, we confirmed the dataset’s relevance by correlating known metabolites with specific bioenergetic dependencies. As anticipated, lactate, which is commonly associated with glycolytic cells, showed a strong correlation with glycolysis. Similarly, TCA cycle metabolites, such as succinate, citrate, fumarate, and malate, positively correlated with the mitochondrial respiration rates, thereby validating the relevance of this dataset.

The distribution of metabolites indicated that cells with high rates of OXPHOS exhibited elevated levels of long, unsaturated phosphatidylcholines and unsaturated cholesteryl esters, which are vital for membrane fluidity and optimal mitochondrial respiration. Furthermore, such cells displayed higher concentrations of propionylcarnitine and lauroylcarnitine, both involved in FAO. Notably, they also demonstrated an increased abundance of carbohydrates, a majority of amino acids, TCA cycle intermediates, and various vitamins, suggesting a preference for substrate accumulation over rapid proliferation. Additionally, these cells also exhibited elevated levels of cAMP, a secondary messenger controlling numerous pathways via PKA kinase, including the regulation of mitochondrial functions [42].

In contrast, glycolytic cells showed a distinct lipid profile, favouring short and saturated phosphatidylcholines and cholesteryl esters essential for rapid membrane synthesis in fast-proliferating cells. These cells also had increased levels of short and saturated sphingomyelins and lysophosphatidylcholines, crucial for lipid metabolism and signaling. Additionally, glycolytic cells exhibited an altered carnitine profile, marked by higher levels of hexanoylcarnitine, valerylcarnitine, and heptanoylcarnitine.

Interestingly, glycolytic cells showed elevated levels of metabolites critical for signaling, metabolism, and carcinogenesis. Notably, alongside upregulated glycolysis, these cells displayed increased NADP, produced via the pentose phosphate pathway and closely linked with glycolysis. NADP and its reduced form, NADPH, are vital for rapidly proliferating cells, supporting fatty acid and nucleotide synthesis, and providing protection from oxidative stress. Surprisingly, malondialdehyde, a marker of oxidative stress and lipid peroxidation associated with high ROS levels, was elevated in the glycolytic but not in the OXPHOS-dependent cells. Additionally, glycolytic cells accumulated 2-hydroxyglutarate (2-HG), an “oncometabolite” known for its role in cancer [43]. While mutations in IDH1 and IDH2 enzymes, typical producers of 2-HG in certain cancers, are rare in CRC, our findings suggest that a glycolytic phenotype may promote the 2-HG accumulation even without these mutations.

The overall picture that emerges from the metabolome of examined cells suggests that cells with high levels of mitochondrial respiration are pointed towards a steady phenotype and efficient energy production. Conversely, cells that favour glycolysis prioritize rapid growth and proliferation, often compromising energy efficiency and potentially increasing vulnerability and stress.

We used PCA and hierarchical/K-means clustering to categorize cell lines by their metabolome, which showed that glycolytic cells and those with high OXPHOS rates tend to cluster together, highlighting a strong link between the bioenergetic profiles and metabolomic characteristics (Fig. 4E, F). We also examined the concordance between the OCR/ECAR-derived clusters and PCA-based cell stratification using the bioenergetic/mitochondrial phenotypes and transcriptomic data, which reflects the overall cell phenotype (Fig. 4G, H). Both analyses indicated that grouping cells by their OCR/ECAR ratios mirrors the stratification performed using other parameters, suggesting that this ratio is a reliable metric for classifying CRC cells by functional phenotypes. The primary metabolite patterns in these groups are summarized in Fig. 4I.

### Efficacy of treatments targeting metabolism and homeostasis is linked to bioenergetics, mitochondrial parameters and metabolite levels

Given the significant differences in bioenergetics, metabolome, and mitochondrial parameters among the CRC cell lines tested, we posited that the efficacy of anti-cancer treatment might vary based on these factors. We hypothesized that cells with high OXPHOS levels might be more susceptible to mitochondrial targeting, while those with high glycolysis could be particularly vulnerable to glycolytic inhibition. To test these hypotheses, we used 18 compounds targeting mitochondrial and metabolic processes, along with conventional anti-CRC chemotherapy (Fig. S6). We then analysed changes in cell number, proliferation, apoptosis, and MMP across the cell lines after 72 h (Figs. S7–S10). By calculating the correlation matrices, we identified associations between the treatment responses and baseline bioenergetic, mitochondrial, and metabolite profiles (Fig. S11).

**Fig. 5:**
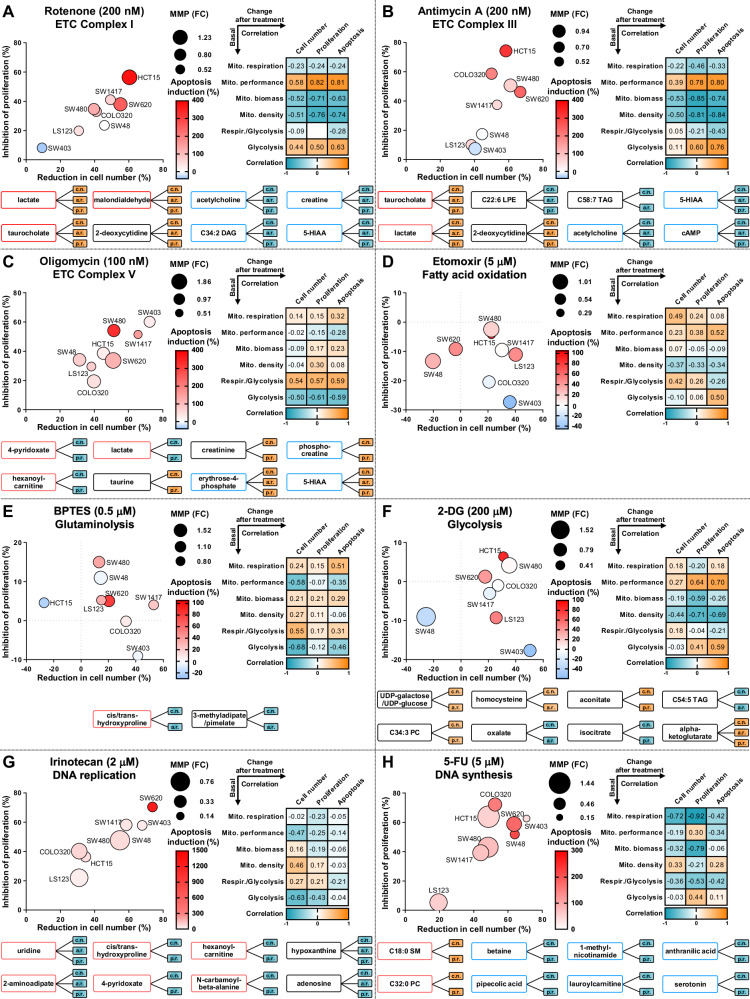
Correlating drug efficacy with bioenergetics, mitochondrial parameters and metabolite levels in CRC cells. **A–H** CRC cells were seeded one day prior to the beginning of the experiment and then treated with selected drugs in indicated concentrations for 72 h. Subsequently, analysis of cell number, apoptosis levels, proliferation rate and MMP was carried out (Figs. S7–S10). The obtained data was quantified and represented as fold changes (FC) or percentages over the control condition. **A–H** **Left side:** Bubble plots illustrate the mean changes in cell number (*x*-axis, %), mean changes in proliferation rate (*y*-axis, %), mean changes in apoptosis (colour, %) and mean changes in MMP (size, fold change). The changes in cell number, proliferation rate, apoptosis levels, and MMP were quantified using flow cytometry along with CellTrace Violet, annexin V, and JC-1 stainings, respectively. **A–H** **Right side:** Correlation matrices depict the relationships between various mitochondrial and bioenergetic characteristics measured at the steady level and the drug responses induced by treatments with selected compounds (see Fig. S11). The drug response is represented by changes in cell number, proliferation rate, and apoptosis. **A–H** **Bottom:** The correlations between the abundance of metabolites and the drug response parameters triggered by each specific compound in the panel of CRC cells (Details continue in Fig. S12A). Each line between a metabolite and a response parameter depicts a strong correlation (<−0.6, blue-filled box; > 0.6, orange-filled box). Only metabolites displaying at least two simultaneous strong correlations are depicted. The drug response is represented by the three parameters change in cell number (c.n.), apoptosis rate (a.r.) and proliferation rate (p.r). The colour of the frames depicts whether metabolites are accumulated in cells with specific bioenergetic profiles. The legends are explained in detail in Fig. S12A.

Intriguingly, treatments targeting ETC complexes displayed variable efficacy based on differing bioenergetic and mitochondrial profiles. Specifically, rotenone and antimycin A were highly effective against cells with high glycolysis and mitochondrial performance (OXPHOS normalized to mitochondrial biomass) but low mitochondrial biomass and density (Fig. 5A, B). In contrast, oligomycin proved more effective in cells with high OCR/ECAR ratios and low glycolysis (Fig. 5C). This observation aligns with metabolite associations where increased lactate levels correlated positively with the efficacy of rotenone and antimycin A but showed a negative correlation with oligomycin (Fig. 5A–C and S12A). These results confirm the increased effectiveness of the ETC Complex I and III inhibitors against glycolytic cells. Additionally, malondialdehyde, an oxidative stress marker, was more prevalent in glycolytic cells and positively associated with the response to rotenone, suggesting a link between Complex I inhibition and ferroptosis induction.

We next examined the main metabolic processes fuelling the TCA cycle, specifically FAO, glutaminolysis, and pyruvate import, targeted by etomoxir, BPTES, and UK5099, respectively. As anticipated, the effectiveness of etomoxir correlated with mitochondrial respiration and OCR/ECAR ratios (Fig. 5D). Similarly, BPTES showed positive correlations with mitochondrial respiration and OCR/ECAR ratios, but negative associations with glycolysis (Fig. 5E). However, the inhibition of pyruvate-specific import by UK5099 showed no significant correlations with mitochondrial respiration (Fig. S12B). These findings suggest that while the efficacy of these inhibitors is somewhat connected to mitochondrial respiration, bioenergetic profiles may not be the primary determinant of their effectiveness.

We also evaluated compounds targeting mitochondrial homeostasis. We used CCCP to induce mitochondrial depolarization, mdivi-1 to disrupt mitochondrial fusion/fission dynamics, cyclosporine A (CsA) to block the mitochondrial permeability transition pore, and dimethyl fumarate to elevate cellular fumarate levels, which are linked with mitochondrial dysfunction. The effect of CCCP was not strongly correlated with baseline mitochondrial features, whereas mdivi-1 showed significant associations with mitochondrial number and density, indicating increased sensitivity in cells with fewer mitochondria (Figs. S12C and S13A). Interestingly, the effects of CsA and dimethyl fumarate were modest but in opposite directions: CsA was more effective in cells with higher mitochondrial respiration/biomass and lower glycolysis, while dimethyl fumarate was more effective in cells with lower mitochondrial respiration and higher glycolysis (Fig. S13B, C).

Subsequently, we evaluated the effectiveness of inhibitors targeting glycolysis and lactate export (Figs. 5F and S13D). Interestingly, the efficacy of these compounds correlated more strongly with mitochondrial characteristics than with glycolytic or bioenergetic profiles, showing greater effects on cells with lower mitochondrial density and higher normalized mitochondrial function. Additionally, the efficacy of 2-DG was positively associated with UDP-glucose levels, likely due to its inhibition of glucose-1-phosphate formation crucial for UDP-glucose synthesis. Thus, cells with higher UDP-glucose utilization exhibited increased sensitivity to glycolytic inhibition.

Glycolysis and mitochondrial respiration are key drivers of intracellular acidification, which, if disrupted, can lead to excessive H^+^ accumulation and cell death [44]. Treatments with EIPA and topiramate, which disrupt cellular pH regulation, were notably more effective in cells with high OXPHOS levels and OCR/ECAR ratios, indicating that mitochondrial activity is a crucial determinant of their efficacy (Fig. S13E, F). On the other hand, concanamycin A, which impairs endosome and lysosome acidification, showed a strong association with reduced mitochondrial respiration and biomass, and increased glycolysis (Fig. S13G).

Finally, we explored the relationship between mitochondrial and bioenergetic metrics and the effectiveness of conventional chemotherapy agents in CRC, analysing responses to irinotecan, oxaliplatin, and 5-fluorouracil (5-FU), (Fig. 5G, H and S13H). While the effectiveness of oxaliplatin showed weak correlation with bioenergetic characteristics, irinotecan and 5-FU demonstrated contrasting efficacy patterns; irinotecan was more effective against cells with low glycolytic activity, whereas 5-FU performed better in cells with reduced OXPHOS levels. Furthermore, the effectiveness of these DNA-targeting therapeutics strongly correlated with metabolites such as uridine, hypoxanthine, adenosine, and thiamine, involved in nucleic acid homeostasis, indicating their suitability as potential biomarkers of therapeutic efficacy.

Our analysis indicates that neither glycolytic cells nor those with high OXPHOS levels exhibit a consistent change in sensitivity to pharmacological treatments (Fig. S14A–D), challenging the prevailing view that glycolytic cancer cells are generally more susceptible to chemotherapeutic agents [45, 46]. However, we noted a greater variability in drug responses, such as changes in cell number, proliferation, apoptosis, and MMP, particularly in more glycolytic cell lines like SW620, SW48, COLO-320, and HCT-15. This variability suggests that glycolytic cells may have a wider range of potential vulnerabilities that require selective targeting.

Additionally, our findings indicate that mitochondrial depolarization, characterized by a drop in MMP, is not consistently linked to apoptosis, proliferation inhibition, or cell death. For example, in SW480 cells, changes in cell number were not correlated with MMP changes. Similarly, in SW620, SW403, and SW48 cells, changes in cell numbers did not align proportionally with MMP alterations. Moreover, treatments specifically targeting mitochondria did not demonstrate the enhanced induction of mitochondrial depolarization compared to conventional chemotherapy agents. Both classes of compounds induced similar changes in cell number, yet 5-FU, irinotecan, and oxaliplatin affected MMP as much as or even more than mitochondrial-specific ETC-targeting inhibitors, such as rotenone, antimycin A, and oligomycin.

### Cancer stratification by mitochondrial, bioenergetic, and metabolic characteristics, is crucial for the high efficacy of metabolism-targeting therapies

**Fig. 6:**
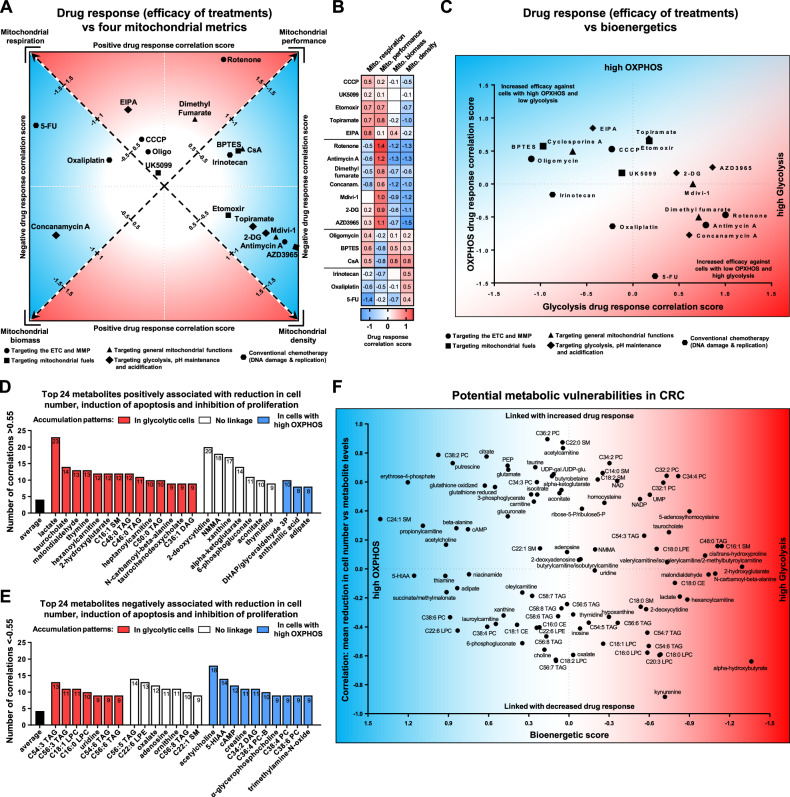
Linking mitochondrial and metabolic traits to therapy efficacy in CRC. **A**, **B** Panels illustrating the relationships between the mitochondrial characteristics of CRC cells and the efficacy of selected treatments in inducing a drug response (changes in cell number, proliferation, and apoptosis) expressed as a drug response correlation score. A positive drug response correlation score indicates that the drug response is more intense in alignment with the specific mitochondrial characteristic (similar to a positive correlation). Conversely, a negative score suggests that the drug response decreases as mitochondrial characteristics are upregulated (similar to a negative correlation). The data is derived from correlation matrices shown in the corresponding Figs. 5, S12, and S13. **A** The diagram highlights the most prominent associations between drug responses and mitochondrial characteristics. **B** The heatmap displays the scores for all four examined mitochondrial characteristics. **C** Graph illustrating the relationship between the OXPHOS/glycolysis levels of CRC cells and the efficacy of selected treatments in inducing a drug response, expressed as a drug response correlation score. **D**, **E** Metabolites with the highest number of strong correlations (<−0.55 or > 0.55) characterizing the linkage between the abundance of these metabolites and the drug response parameters (changes in cell number, proliferation rate and apoptosis). The colours of the bars indicate whether metabolites accumulate in cells with specific bioenergetic profiles. **F** Graph illustrating the relationships between the abundance of metabolites in CRC cells and the mean sensitivity of these cells to the examined treatments. The *X*-axis represents the bioenergetic score, where higher values indicate greater accumulation of the metabolite in cells with increased OXPHOS, and negative values indicate greater accumulation in cells with high glycolysis. The *Y*-axis depicts correlation values showing the linkage between the abundance of metabolites and the average change in cell number upon treatment with 18 compounds. As a result, metabolites in the upper right part of the graph are prone to accumulate in glycolytic cells and are potentially associated with increased drug responses. Conversely, metabolites in the bottom left part of the graph tend to accumulate in cells with high OXPHOS and might be associated with decreased sensitivity to drugs.

To quantify the relationship between the changes in cell number, proliferation, and apoptosis, with baseline mitochondrial or bioenergetic metrics, we developed a drug response correlation score. This score, described in detail in the Materials and Methods section and shown in Fig. S15A, aggregates three weighted Pearson correlation coefficients. A positive score indicates an increase in drug response correlating with an increase in a mitochondrial characteristic, and conversely, a negative score indicates a decrease in drug response as mitochondrial characteristics intensify. We used this score to identify which mitochondrial parameters—respiration, biomass, density, and performance—show the strongest associations with each treatment (Fig. 6A).

Treatments with etomoxir, topiramate, mdivi-1, antimycin A, 2-DG, and AZD3965 showed the strongest associations with mitochondrial density, thereby demonstrating greater efficacy against cells with reduced mitochondrial density. Conversely, rotenone and EIPA exhibited higher effectiveness in cells with elevated normalized mitochondrial performance and absolute mitochondrial respiration, respectively. The heatmap illustrating the distribution of these scores among evaluated mitochondrial characteristics identifies four main association patterns (Fig. 6B). Notably, the most distinct cluster consists of rotenone, antimycin A, dimethyl fumarate, concanamycin A, mdivi-1, 2-DG, and AZD3965, where an increase in treatment efficacy corresponded with both high baseline mitochondrial performance and low mitochondrial biomass/density.

To identify the bioenergetic sensitivities of CRC cell lines, we analysed drug response scores in relation to baseline OXPHOS and glycolysis levels (Fig. 6C). The analysis showed that treatments targeting mitochondria, including inhibitors and modulators of MPTP opening, Complex V functionality, glutaminolysis, FAO, and MMP, were more effective in cells with high OXPHOS levels. In contrast, cells with a glycolytic bioenergetic profile were more susceptible to inhibitors of ETC Complexes I and III, highlighting the critical nature of their residual activity for CRC cell survival.

To guide targeted therapies, we analysed the associations between metabolite levels and treatment outcomes such as changes in cell number, apoptosis, and proliferation (Fig. 6D). On average, metabolites showed four strong correlations with drug response parameters. Notably, lactate, 2-deoxycytidine, NMMA, and xanthine demonstrated a higher number of positive correlations, suggesting that elevated levels might enhance responsiveness to treatments or indicate vulnerability. Conversely, various fatty acids and metabolites like acetylcholine, 5-HIAA, and cAMP negatively correlated with drug response (Fig. 6E). As potent signaling mediators, their increased levels might confer protective properties to cells, making them potential targets for adjuvant therapy.

To further develop this idea, we examined whether the levels of the top 96 metabolites, most associated with drug response, consistently correlated with cellular susceptibility to treatments. We calculated the average change in cell number for each cell line across all 18 treatments and correlated these changes with the basal metabolite levels in each cell line (see Fig. S15B). Additionally, we plotted cell number response values against a bioenergetic score, which reflects each metabolite’s affinity to OXPHOS or glycolysis, to link metabolite levels to both drug response and baseline bioenergetic characteristics (see Fig. S15C).

This analysis demonstrated that higher concentrations of choline, oxalate, kynurenine, alpha-hydroxybutyrate, long-chain polyunsaturated triacylglycerols, and lysophosphatidylcholines correlated with reduced changes in cell numbers after treatment, suggesting that these compounds may provide protective properties or indicate a stable and resilient cellular phenotype (Fig. 6F). In contrast, elevated levels of erythrose-4-phosphate, putrescine, citrate, glutathione, taurine, 2-phosphoenolpyruvate, glutamate, butyrobetaine, UDP-galactose/glucose, NAD, acetylcarnitine, and certain lipids, including specific sphingomyelins and phosphatidylcholines (e.g., C38:2, C36:2, C34:2, C32:2, C34:2), were linked to increased drug efficacy. Correlations between the abundance of these metabolites and the efficacy of individual treatments are detailed in Fig. S16A.

Collectively, the conducted analysis demonstrates that both OXPHOS-dependent and glycolytic CRC phenotypes are characterized by vulnerabilities associated with the distribution of different metabolites. We also identified sets of metabolites that show strong correlations with specific drug response parameters in CRC cells (Fig. S16B–D). Hypothetically, metabolites whose levels negatively correlate with drug response parameters may inhibit changes in proliferation, apoptosis, and MMP, whereas those that positively correlate with drug response parameters could enhance changes in these parameters.

## Discussion

The aim of this study was to explore the level of mitochondrial and bioenergetic heterogeneity in different CRC cells and to determine if this heterogeneity can be correlated with the effectiveness of metabolism-targeting therapies. We observed a major level of variability in the mitochondrial biomass, the mitochondrial protein density, and the mitochondrial composition between the cell lines analysed. Despite similar expression levels of nucleus-encoded mitochondrial transcripts across the cell lines tested, some of them showed markedly reduced mitochondrial biomasses, suggesting the influence of potent mechanisms that affect the number of mitochondria at post-transcriptional levels.

Furthermore, contrary to expectations, a higher abundance of mitochondrial proteins did not always correlate with increased mitochondria quantities. Instead, CRC cells with low mitochondrial biomasses displayed a high concentration of mitochondrial proteins, indicating fewer mitochondria with increased protein density and high functional capacity. This suggests that mitochondria in these cancer cells may function as a “molecular sponge,” absorbing and integrating mitochondrial proteins efficiently without necessarily increasing in number or biomass. This feature is supported by studies indicating substantial variability in mitochondrial protein density [47, 48].

The proteomics analysis of 453 mitochondrial proteins uncovered significant structural and functional heterogeneity among the mitochondria studied. They displayed major differences in the abundance of proteins involved in the ETC, TCA cycle, OXPHOS, and the metabolism of amino acids and fatty acids, as well as apoptosis machinery, indicating a high level of specialisation. Additionally, we filtered out proteins that are currently not classified as mitochondrial according to existing databases. However, some proteins were consistently present in various mitochondrial samples, suggesting that they could be associated with these organelles in cancer.

Along with significant variability in the mitochondrial composition, the functional stratification of CRC cells revealed the presence of distinct mitochondrial and bioenergetic strategies that drive cell sustainability. However, it is clear that this sample size is not sufficient to draw general conclusions about the role of mitochondria and their plasticity in cancer. Our knowledge of the mitochondrial proteome in cancer cells remains limited. This area of research necessitates extensive mitochondria-targeting proteomic studies that employ both cell lines and cells isolated directly from primary and metastatic tumours. Such research would help define specific mitochondrial archetypes and their functions in cancer.

The comprehensive evaluation of mitochondria in cancer cells has not been feasible previously without the omics technologies owing to the vast number of genes encoding these organelles [49–51]. Furthermore, current omics studies have been dominated by transcriptomic approaches, which are not very effective for mitochondrial analysis. In cancer cells, the transcriptomic RNA levels only modestly correlate with the abundance of mitochondrial proteins [52, 53]. This discrepancy occurs because mitochondrial proteins do not exist in isolation; they exist and are functional when integrated into larger organelle structures. Therefore, the abundance of mitochondrial proteins is affected by different factors, including mitochondrial turnover, dynamics between biogenesis and mitophagy, translation of mitochondrial proteins, import of proteins into mitochondria, as well as the assembly of mitochondrial complexes and structures [54–56]. These factors can lead to impaired mitochondrial assembly and various perturbations, thus creating a significant divergence between the abundance of RNA and proteins.

Considering the observed heterogeneity, we connected two areas of cancer biology research currently receiving significant attention: cancer cell metabolic adaptations and the potential use of drugs that target metabolic pathways as cancer treatment. We hypothesized that the efficacy of these compounds would depend on the mitochondrial and metabolic status of the cells. To validate this, we treated CRC cells with 18 compounds targeting various mitochondrial, metabolic and related homeostatic processes and examined their efficacies.

The efficacy of most treatments showed a notable yet not uniformed correlation with cellular bioenergetics. For example, inhibiting ETC Complexes I and III had a more pronounced effect on CRC cells reliant on glycolysis compared to those dependent on OXPHOS. Surprisingly, 2-DG, a glycolysis inhibitor, did not exhibit increased cytotoxicity in cells more dependent on glycolysis. However, the effectiveness of these compounds often closely aligned with mitochondrial characteristics, indicating that mitochondrial status significantly reflects the overall cellular condition. Given that mitochondria are central to numerous cellular processes and are also instrumental in the onset of apoptosis, it is unsurprising that the efficacy of many treatments strongly correlates with mitochondrial parameters.

Additionally, we identified metabolites whose levels were strongly linked to the magnitude of the drug response. These results suggest that certain metabolites may serve as biomarkers for metabolic vulnerabilities in CRC. Co-targeting metabolic pathways involved in the conversion of these metabolites may yield synergistic effects. For example, the efficacy of irinotecan and oxaliplatin treatments inversely correlated with adenosine levels, indicating that inhibiting the salvage or de novo synthesis of purine nucleotides could enhance the effectiveness of DNA-targeting treatments.

Furthermore, the levels of some metabolites strongly correlated with the efficacy of different treatments, suggesting that these molecules might be essential for cell survival. We categorized these metabolites by their accumulation profiles in cells with either glycolytic or OXPHOS dependencies. Using this approach, we revealed the existence of distinct metabolic vulnerabilities linked to bioenergetic preferences. For instance, kynurenine, primarily accumulating in glycolytic cells, was associated with a reduced treatment response. Numerous studies confirm that kynurenine supports CRC cell viability and promotes cancer progression and chemoresistance [57–59].

Metabolic anti-cancer therapies which starve cancer cells of asparagine, antifolates, and inhibitors of isocitrate dehydrogenases, preventing the synthesis of “oncometabolites” are already available [60]. Moreover, dozens of new metabolism-targeting agents are currently being evaluated in clinical trials, including those depleting arginine and inhibiting glycolysis [61, 62]. Our study clearly demonstrates that these therapies should be personalized, because in many cases the drug response to these treatments is heavily linked to the mitochondrial, bioenergetic and metabolic state of cells. Therefore, clinical trials require initial patient stratification based on the metabolic activity of cells.

## Supplementary information

Suppl. Figures 1-16

Dataset 1

Dataset 2

Dataset 3

Dataset 4

Dataset 5

Dataset 6

Dataset 7

original immunoblots

## Data Availability

All data are available in the main text or the supplementary materials.
