# Supplementary material for: Mitochondrial, metabolic and bioenergetic adaptations drive plasticity of colorectal cancer cells and shape their chemosensitivity
Source: Cell Death Dis. 2025 Apr 5;16(1):253. doi: 10.1038/s41419-025-07596-y (PMC11971274; doi:10.1038/s41419-025-07596-y)
Supplement: Supplementary file 1 — Suppl. Figures 1-16 [file 41419_2025_7596_MOESM1_ESM.pdf]

Supplementary Materials for

Mitochondrial, Metabolic and Bioenergetic Adaptations Drive Plasticity of Colorectal Cancer Cells and Shape Their Chemosensitivity

Nikita Markov *et al.*

*Corresponding author: Hans-Uwe Simon, hans-uwe.simon@unibe.ch

This PDF file includes:

Figures and figure legends S1 to S16
Legends for data files S1 to S7

Other Supplementary Materials for this manuscript include the following:

Data files S1 to S7
Raw WB

Fig.S1

A

Characteristics and features of 8 CRC cell lines

Name	TP53	KRAS	BRAF	PIK3CA	PTEN	MSI	CIMP	CMS	Origin	Type/Grade	Sex	Source	Collection site
SW480	p.R273H, p.P309S	p.G12V	wt	wt	wt	MSS	CIMP-	CMS4	epithelial	Dukes' C/-	M	Primary	Colon
SW1417	p.C238fs	wt	p.V600E	wt	wt	MSS	CIMP-	CMS2	epithelial	Dukes' C/III	F	Primary	Colon
LS123	p.R175H	p.G12S	wt	wt	p.R208H	MSS	CIMP-	CMS4	epithelial	-/-	F	Primary	Colon
SW403	p.E51X	p.G12V	wt	wt	wt	MSS	CIMP-	CMS2	epithelial	Dukes' C/III	F	Primary	Colon
SW620	p.R273H, p.P309S	p.G12V	wt	wt	wt	MSS	CIMP-	CMS4	epithelial	Dukes' C/-	M	Metastasis	Lymph node
SW48	wt	wt	p.R347X	p.G914R	wt	MSI	CIMP+	CMS1	epithelial	Dukes' C/IV	F	Primary	Colon
COLO-320	p.R248W	wt	wt	wt	wt	MSS	CIMP-	-	neuroendocrine	-/-	F	Primary	Colon
HCT-15	p.S241F	p.G13D	wt	p.E545K, p.D549N	wt	MSI	CIMP+	CMS1	epithelial	Dukes' C	M	Primary	Colon

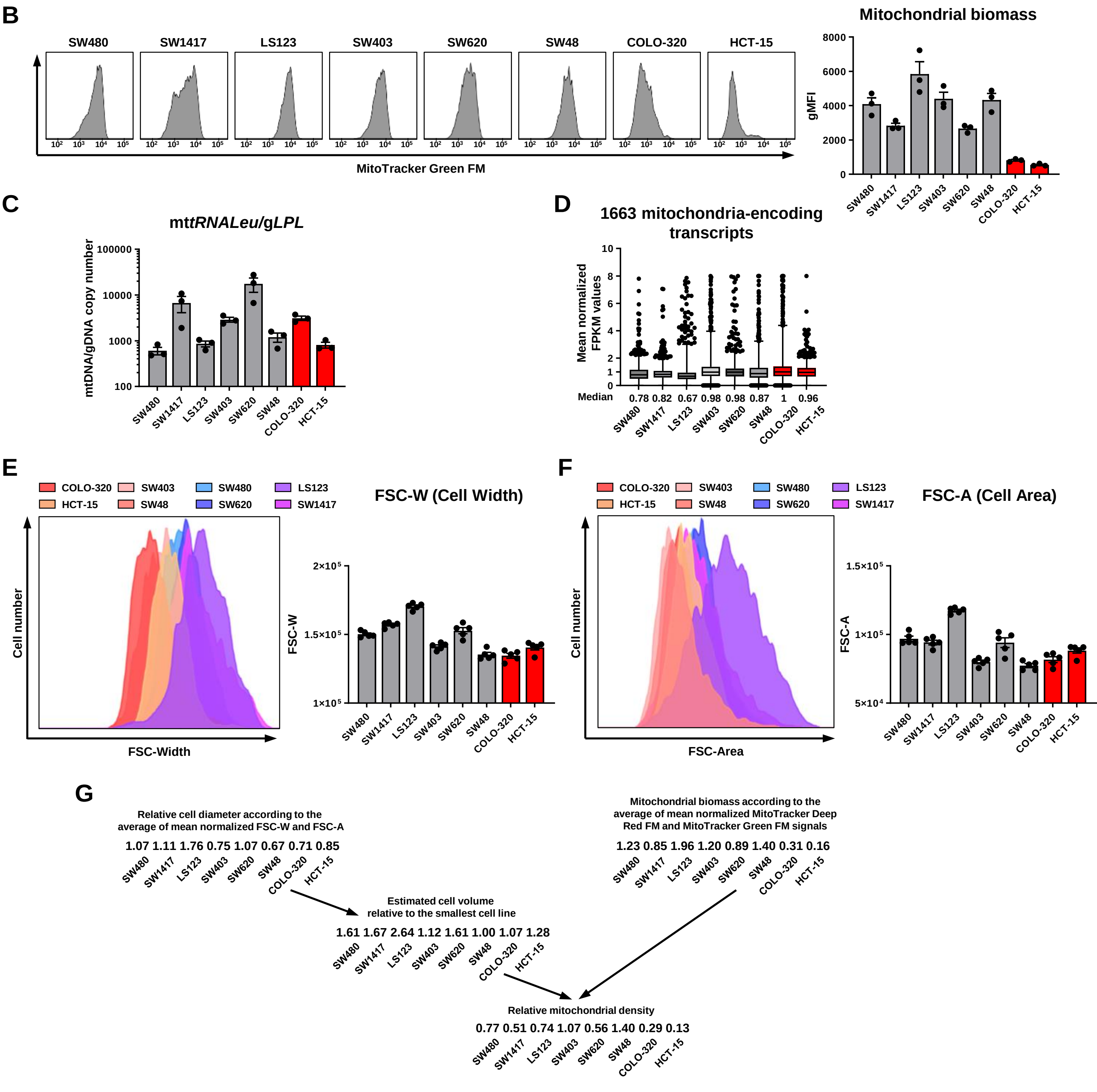

Supplementary Figure 1. General Features and Mitochondrial Characteristics of CRC Cell Lines. (**A**) Key characteristics of examined CRC cell lines. (**B**) Representative histograms and their quantification depicting mitochondrial biomass assessed by flow cytometry analysis of CRC cells stained with 200 nM MitoTracker Green FM dye. Values are means ± SEM, n = 3. (**C**) qPCR-quantified mtDNA copy number normalized against genomic copy number. Values are means ± SEM, n = 3. (**D**) Mean normalized FPKM values of 1663 nuclear transcripts encoding mitochondrial proteins. Each dot represents one gene, with whiskers representing the 2.5-97.5 percentile. The data extracted from the CCLE project. (**E-F**) Quantification and representative plots of flow cytometry analysis of cell size (diameter) using FSC-W and FSC-A scatters reflecting cell width and area, respectively. Values are medians ± SEM, n = 5. (**G**) Calculation of cell volume and relative mitochondrial density based on the measured cell size and the absolute mitochondria abundance.

Fig.S2

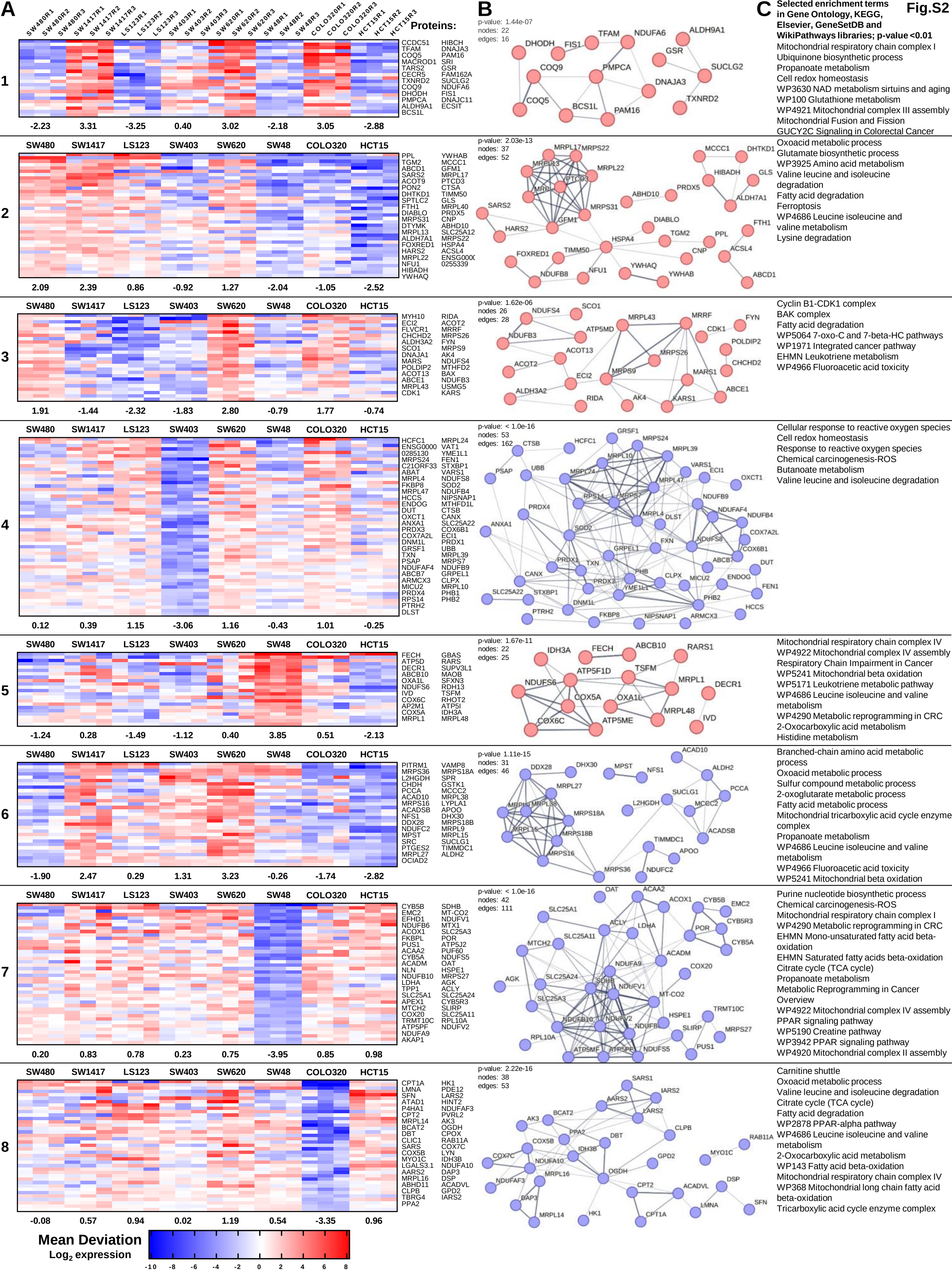

A
B
C
SW480R1 SW480R2 SW480R3 SW1417R1 SW1417R2 SW1417R3 LS123R1 LS123R2 LS123R3 SW403R1 SW403R2 SW403R3 SW620R1 SW620R2 SW620R3 SW48R1 SW48R2 SW48R3 COLO320R1 COLO320R2 COLO320R3 HCT15R1 HCT15R2 HCT15R3
Proteins:
Selected enrichment terms in Gene Ontology, KEGG, Elsevier, GeneSetDB and WikiPathways libraries; p-value <0.01
1
CCDC51 TFAM COQ5 MACROD1 TARS2 CECR5 TXNRD2 COQ9 DHODH PMPCA ALDH9A1 BCS1L
HIBCH DNAJA3 PAM16 SRI GSR FAM162A SUCLG2 NDUFA6 FIS1 DNAJC11 ECSIT
-2.23 3.31 -3.25 0.40 3.02 -2.18 3.05 -2.88
p-value: 1.44e-07
nodes: 22
edges: 16
Mitochondrial respiratory chain complex I
Ubiquinone biosynthetic process
Propanoate metabolism
Cell redox homeostasis
WP3630 NAD metabolism sirtuins and aging
WP100 Glutathione metabolism
WP4921 Mitochondrial complex III assembly
Mitochondrial Fusion and Fission
GUCY2C Signaling in Colorectal Cancer
SW480 SW1417 LS123 SW403 SW620 SW48 COLO320 HCT15
2
PPL TGM2 ABCD1 SARS2 ACOT9 PON2 DHTKD1 SPTLC2 FTH1 DIABLO MRPS31 DTYMK MRPL13 ALDH7A1 FOXRED1 HARS2 MRPL22 NFU1 HIBADH YWHAQ
YWHAB MCCC1 GFM1 MRPL17 PTCD3 CTSA TIMM50 GLS MRPL40 PRDX5 CNP ABHD10 SLC25A12 MRPS22 HSPA4 ACSL4 ENSG0000 0255339
2.09 2.39 0.86 -0.92 1.27 -2.04 -1.05 -2.52
p-value: 2.03e-13
nodes: 37
edges: 52
Oxoacid metabolic process
Glutamate biosynthetic process
WP3925 Amino acid metabolism
Valine leucine and isoleucine degradation
Fatty acid degradation
Ferroptosis
WP4686 Leucine isoleucine and valine metabolism
Lysine degradation
3
MYH10 ECI2 FLVCR1 CHCHD2 ALDH3A2 SCO1 DNAJA1 MARS POLDIP2 ACOT13 ABCE1 MRPL43 CDK1
RIDA ACOT2 MRRF MRPS26 FYN MRPS9 AK4 NDUFS4 MTHFD2 BAX NDUFB3 USMG5 KARS
1.91 -1.44 -2.32 -1.83 2.80 -0.79 1.77 -0.74
p-value: 1.62e-06
nodes 26
edges: 28
Cyclin B1-CDK1 complex
BAK complex
Fatty acid degradation
WP5064 7-oxo-C and 7-beta-HC pathways
WP1971 Integrated cancer pathway
EHMN Leukotriene metabolism
WP4966 Fluoroacetic acid toxicity
4
HCFC1 ENSG0000 0285130 MRPS24 C21ORF33 ABAT MRPL4 FKBP8 MRPL47 HCCS ENDOG DUT OXCT1 ANXA1 PRDX3 COX7A2L DNM1L GRSF1 TXN PSAP NDUFAF4 ABCB7 ARMCX3 MICU2 PRDX4 RPS14 PTRH2 DLST
MRPL24 VAT1 YME1L1 FEN1 STXBP1 VARS1 NDUFS8 SOD2 NDUFB4 NIPSNAP1 MTHFD1L CTSB CANX SLC25A22 COX6B1 ECI1 PRDX1 UBB MRPL39 MRPS7 NDUFB9 GRPEL1 CLPX MRPL10 PHB1 PHB2
0.12 0.39 1.15 -3.06 1.16 -0.43 1.01 -0.25
p-value: < 1.0e-16
nodes: 53
edges: 162
Cellular response to reactive oxygen species
Cell redox homeostasis
Response to reactive oxygen species
Chemical carcinogenesis-ROS
Butanoate metabolism
Valine leucine and isoleucine degradation
5
FECH ATP5D DECR1 ABCB10 OXA1L NDUFS6 IVD COX6C AP2M1 COX5A MRPL1
GBAS RARS SUPV3L1 MAOB SFXN3 RDH13 TSFM RHOT2 ATP5I IDH3A MRPL48
-1.24 0.28 -1.49 -1.12 0.40 3.85 0.51 -2.13
p-value: 1.67e-11
nodes: 22
edges: 25
Mitochondrial respiratory chain complex IV
WP4922 Mitochondrial complex IV assembly
Respiratory Chain Impairment in Cancer
WP5241 Mitochondrial beta oxidation
WP5171 Leukotriene metabolic pathway
WP4686 Leucine isoleucine and valine metabolism
WP4290 Metabolic reprogramming in CRC
2-Oxocarboxylic acid metabolism
Histidine metabolism
6
PITRM1 MRPS36 L2HGDH CHDH PCCA ACAD10 MRPS16 ACADSB NFS1 DDX28 NDUFC2 MPST SRC PTGES2 MRPL27 OCIAD2
VAMP8 MRPS18A SPR GSTK1 MCCC2 MRPL38 LYPLA1 APOO DHX30 MRPS18B MRPL9 MRPL15 SUCLG1 TIMMDC1 ALDH2
-1.90 2.47 0.29 1.31 3.23 -0.26 -1.74 -2.82
p-value 1.11e-15
nodes: 31
edges: 46
Branched-chain amino acid metabolic process
Oxoacid metabolic process
Sulfur compound metabolic process
2-oxoglutarate metabolic process
Fatty acid metabolic process
Mitochondrial tricarboxylic acid cycle enzyme complex
Propanoate metabolism
WP4686 Leucine isoleucine and valine metabolism
WP4966 Fluoroacetic acid toxicity
WP5241 Mitochondrial beta oxidation
7
CYB5B EMC2 EFHD1 NDUFB6 ACOX1 FKBPL PUS1 ACAA2 CYB5A ACADM NLN NDUFB10 LDHA TPP1 SLC25A1 APEX1 MTCH2 COX20 TRMT10C ATP5PF NDUFA9 AKAP1
SDHB MT-CO2 NDUFV1 MTX1 SLC25A3 POR ATP5J2 PUF60 NDUFS5 OAT HSPE1 MRPS27 AGK ACLY SLC25A24 CYB5R3 SLIRP SLC25A11 RPL10A NDUFV2
0.20 0.83 0.78 0.23 0.75 -3.95 0.85 0.98
p-value: < 1.0e-16
nodes: 42
edges: 111
Purine nucleotide biosynthetic process
Chemical carcinogenesis-ROS
Mitochondrial respiratory chain complex I
WP4290 Metabolic reprogramming in CRC
EHMN Mono-unsaturated fatty acid beta-oxidation
EHMN Saturated fatty acids beta-oxidation
Citrate cycle (TCA cycle)
Propanoate metabolism
Metabolic Reprogramming in Cancer Overview
WP4922 Mitochondrial complex IV assembly
PPAR signaling pathway
WP5190 Creatine pathway
WP3942 PPAR signaling pathway
WP4920 Mitochondrial complex II assembly
8
CPT1A LMNA SFN ATAD1 P4HA1 CPT2 MRPL14 BCAT2 DBT CLIC1 SARS COX5B MYO1C LGALS3.1 AARS2 MRPL16 ABHD11 CLPB TBRG4 PPA2
HK1 PDE12 LARS2 HINT2 NDUFAF3 PVRL2 AK3 OGDH CPOX RAB11A COX7C LYN IDH3B NDUFA10 DAP3 DSP ACADVL GPD2 IARS2
-0.08 0.57 0.94 0.02 1.19 0.54 -3.35 0.96
p-value: 2.22e-16
nodes: 38
edges: 53
Carnitine shuttle
Oxoacid metabolic process
Valine leucine and isoleucine degradation
Citrate cycle (TCA cycle)
Fatty acid degradation
WP2878 PPAR-alpha pathway
WP4686 Leucine isoleucine and valine metabolism
2-Oxocarboxylic acid metabolism
WP143 Fatty acid beta-oxidation
Mitochondrial respiratory chain complex IV
WP368 Mitochondrial long chain fatty acid beta-oxidation
Tricarboxylic acid cycle enzyme complex
Mean Deviation
Log2 expression
-10 -8 -6 -4 -2 0 2 4 6 8

Supplementary Figure 2. Functional Insights into Mitochondrial Protein Variability Across CRC Cell Lines. (**A-C**) Label-free LC-MS/MS proteomic analysis of mitochondria extracted from three biological replicates across 8 CRC cell lines. Only 273 of the most variable proteins were used for this analysis. (**A**) K-means clustering of mitochondrial proteins based on their abundance in mitochondria of the examined cells. The number underneath each cluster reflects the mean abundance deviation for each group of proteins relative to the average level. The names of proteins on the right side are listed in top-to-bottom order. (**B**) STRING-based analysis and visualisation of networks corresponding to each cluster defined by K-means analysis. The number of nodes and edges is highlighted at the upper part of each network. Only proteins, exhibiting at least one interaction with other proteins from the same cluster are depicted. (**C**) Enrichment analysis on proteins selected from each corresponding cluster. Only selected enrichment terms are depicted.

Fig.S3

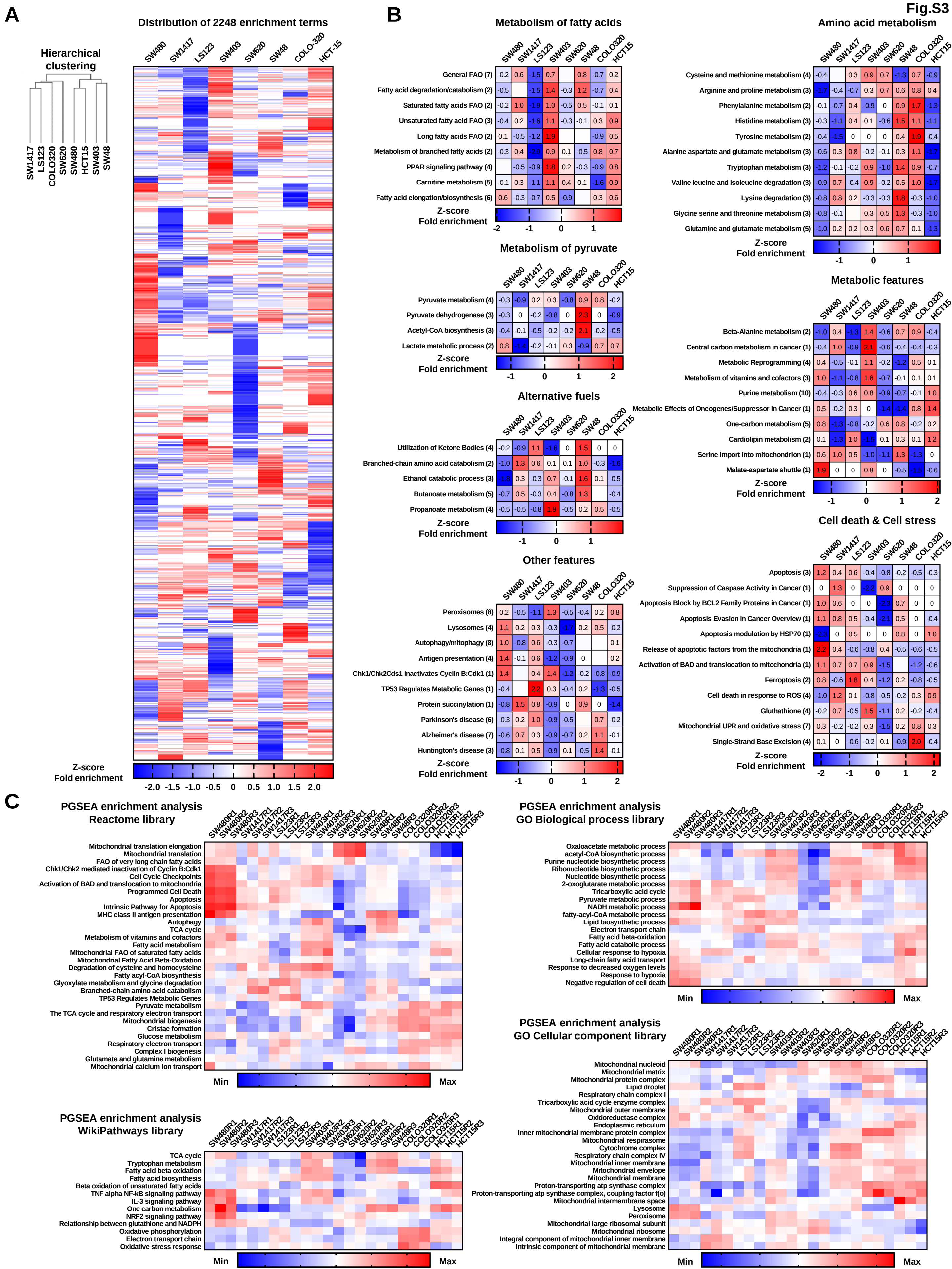
A
Distribution of 2248 enrichment terms
Hierarchical clustering
SW480 SW1417 LS123 SW403 SW620 SW48 COLO-320 HCT-15
SW1417 LS123 COLO320 SW620 SW480 HCT15 SW403 SW48
Z-score Fold enrichment
-2.0 -1.5 -1.0 -0.5 0 0.5 1.0 1.5 2.0
B
Metabolism of fatty acids
SW480 SW1417 LS123 SW403 SW620 SW48 COLO320 HCT15
General FAO (7)
Fatty acid degradation/catabolism (2)
Saturated fatty acids FAO (2)
Unsaturated fatty acid FAO (3)
Long fatty acids FAO (2)
Metabolism of branched fatty acids (2)
PPAR signaling pathway (4)
Carnitine metabolism (5)
Fatty acid elongation/biosynthesis (6)
Metabolism of pyruvate
Pyruvate metabolism (4)
Pyruvate dehydrogenase (3)
Acetyl-CoA biosynthesis (3)
Lactate metabolic process (2)
Alternative fuels
Utilization of Ketone Bodies (4)
Branched-chain amino acid catabolism (2)
Ethanol catabolic process (3)
Butanoate metabolism (5)
Propanoate metabolism (4)
Other features
Peroxisomes (8)
Lysosomes (4)
Autophagy/mitophagy (8)
Antigen presentation (4)
Chk1/Chk2Cds1 inactivates Cyclin B:Cdk1 (1)
TP53 Regulates Metabolic Genes (1)
Protein succinylation (1)
Parkinson's disease (6)
Alzheimer's disease (7)
Huntington's disease (3)
Amino acid metabolism
Cysteine and methionine metabolism (4)
Arginine and proline metabolism (3)
Phenylalanine metabolism (2)
Histidine metabolism (3)
Tyrosine metabolism (2)
Alanine aspartate and glutamate metabolism (3)
Tryptophan metabolism (3)
Valine leucine and isoleucine degradation (3)
Lysine degradation (3)
Glycine serine and threonine metabolism (3)
Glutamine and glutamate metabolism (5)
Metabolic features
Beta-Alanine metabolism (2)
Central carbon metabolism in cancer (1)
Metabolic Reprogramming (4)
Metabolism of vitamins and cofactors (3)
Purine metabolism (10)
Metabolic Effects of Oncogenes/Suppressor in Cancer (1)
One-carbon metabolism (5)
Cardiolipin metabolism (2)
Serine import into mitochondrion (1)
Malate-aspartate shuttle (1)
Cell death & Cell stress
Apoptosis (3)
Suppression of Caspase Activity in Cancer (1)
Apoptosis Block by BCL2 Family Proteins in Cancer (1)
Apoptosis Evasion in Cancer Overview (1)
Apoptosis modulation by HSP70 (1)
Release of apoptotic factors from the mitochondria (1)
Activation of BAD and translocation to mitochondria (1)
Ferroptosis (2)
Cell death in response to ROS (4)
Gluthathione (4)
Mitochondrial UPR and oxidative stress (7)
Single-Strand Base Excision (4)
Z-score Fold enrichment
C
PGSEA enrichment analysis Reactome library
Mitochondrial translation elongation
Mitochondrial translation
FAO of very long chain fatty acids
Chk1/Chk2 mediated inactivation of Cyclin B:Cdk1
Cell Cycle Checkpoints
Activation of BAD and translocation to mitochondria
Programmed Cell Death
Apoptosis
Intrinsic Pathway for Apoptosis
MHC class II antigen presentation
Autophagy
TCA cycle
Metabolism of vitamins and cofactors
Fatty acid metabolism
Mitochondrial FAO of saturated fatty acids
Mitochondrial Fatty Acid Beta-Oxidation
Degradation of cysteine and homocysteine
Fatty acyl-CoA biosynthesis
Glyoxylate metabolism and glycine degradation
Branched-chain amino acid catabolism
TP53 Regulates Metabolic Genes
Pyruvate metabolism
The TCA cycle and respiratory electron transport
Mitochondrial biogenesis
Cristae formation
Glucose metabolism
Respiratory electron transport
Complex I biogenesis
Glutamate and glutamine metabolism
Mitochondrial calcium ion transport
Min Max
PGSEA enrichment analysis WikiPathways library
TCA cycle
Tryptophan metabolism
Fatty acid beta oxidation
Fatty acid biosynthesis
Beta oxidation of unsaturated fatty acids
TNF alpha NF-kB signaling pathway
IL-3 signaling pathway
One carbon metabolism
NRF2 signaling pathway
Relationship between glutathione and NADPH
Oxidative phosphorylation
Electron transport chain
Oxidative stress response
Min Max
PGSEA enrichment analysis GO Biological process library
Oxaloacetate metabolic process
acetyl-CoA biosynthetic process
Purine nucleotide biosynthetic process
Ribonucleotide biosynthetic process
Nucleotide biosynthetic process
2-oxoglutarate metabolic process
Tricarboxylic acid cycle
Pyruvate metabolic process
NADH metabolic process
fatty-acyl-CoA metabolic process
Lipid biosynthetic process
Electron transport chain
Fatty acid beta-oxidation
Fatty acid catabolic process
Cellular response to hypoxia
Long-chain fatty acid transport
Response to decreased oxygen levels
Response to hypoxia
Negative regulation of cell death
Min Max
PGSEA enrichment analysis GO Cellular component library
Mitochondrial nucleoid
Mitochondrial matrix
Mitochondrial protein complex
Lipid droplet
Respiratory chain complex I
Tricarboxylic acid cycle enzyme complex
Mitochondrial outer membrane
Oxidoreductase complex
Endoplasmic reticulum
Inner mitochondrial membrane protein complex
Mitochondrial respirasome
Cytochrome complex
Respiratory chain complex IV
Mitochondrial inner membrane
Mitochondrial envelope
Mitochondrial membrane
Proton-transporting atp synthase complex
Proton-transporting atp synthase complex, coupling factor f(o)
Mitochondrial intermembrane space
Lysosome
Peroxisome
Mitochondrial large ribosomal subunit
Mitochondrial ribosome
Integral component of mitochondrial inner membrane
Intrinsic component of mitochondrial membrane
Min Max

Supplementary Figure 3. Enrichment Analysis of Mitochondrial Proteins across CRC Cell Lines. (**A-C**) Label-free LC-MS/MS proteomic analysis of mitochondria extracted from three biological replicates across 8 CRC cell lines. (**A**) Heatmap depicting Z-transformed fold enrichment values corresponding to the enrichment of 2248 terms from 11 libraries. (**B**) Heatmaps illustrating Z-score values reflecting the average of multiple Z-transformed fold enrichment scores (continuation of Figure 2E). The number in the brackets indicates the number of similar enrichment terms used for the creation of “the consensus enrichment term”. Number 1 reflects that only one original term was used. (**C**) PGSEA-based enrichment analysis was performed on all 24 mitochondrial samples simultaneously. The pathway significance cut-off is 0.2. Only selected enrichment terms are depicted.

Fig.S4

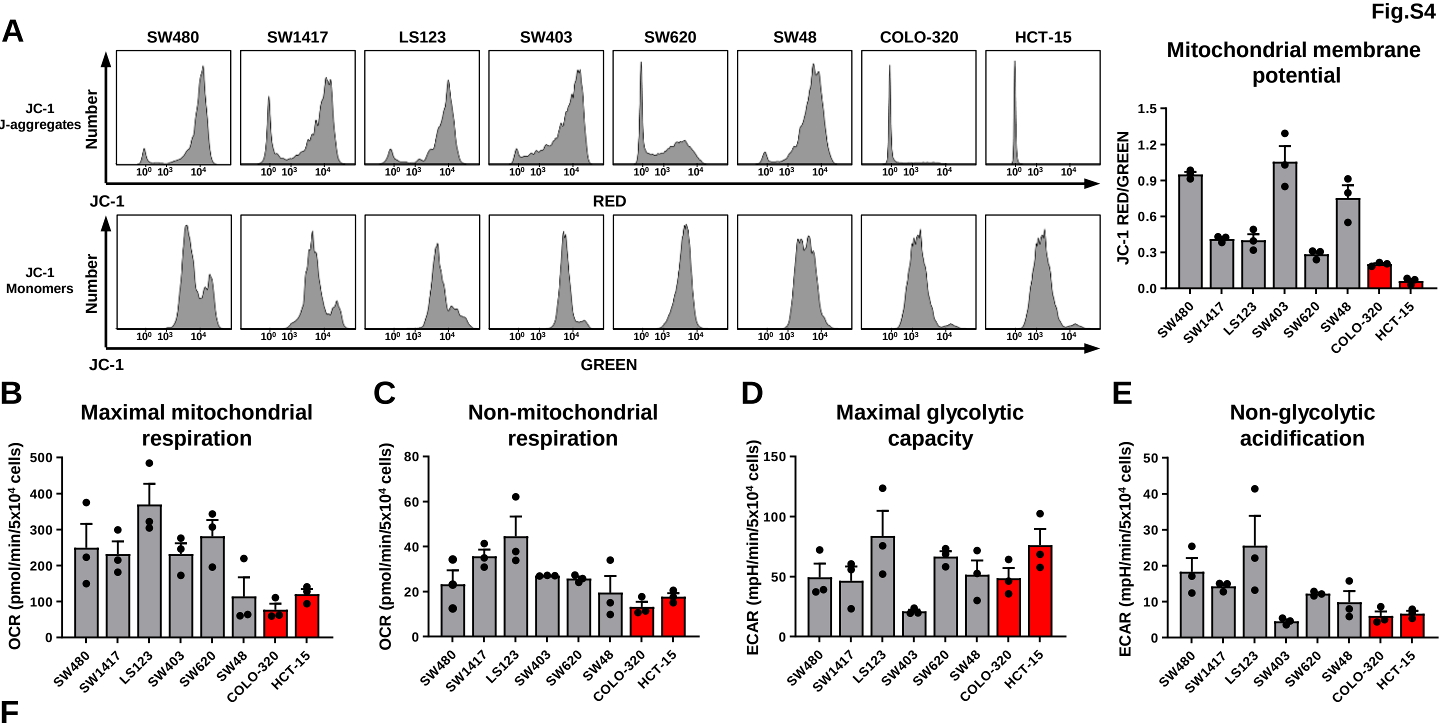

F

Parameter	Description	Values	SW480	SW1417	LS123	SW403	SW620	SW48	COLO-320	HCT-15
**Mitochondrial respiration**	Values derived from the Seahorse bioenergetic analysis	Z-score	0.29	0.76	1.91	-0.27	0.09	-0.82	-1.05	-0.90
**Mitochondrial performance**	Mitochondrial respiration divided by mitochondrial biomass, relative to SW48	Mean normalized	2.12	3.68	2.25	1.66	2.69	1.00	3.70	7.91
**Mitochondrial biomass**	Average of Z-transformed MitoTracker Deep Red FM and MitoTracker Green FM signals	Z-score	0.40	-0.25	1.62	0.34	-0.19	0.67	-1.18	-1.42
**Mitochondrial density**	Mitochondrial biomass normalized to the cell volume, relative to HCT-15	Mean normalized	5.97	3.98	5.77	8.31	4.32	10.86	2.24	1.00
**Respiration/Glycolysis ratio**	Mitochondrial respiration divided by glycolysis (OCR/ECAR ratio)	Z-score	0.77	2.00	0.26	-0.13	-0.35	-0.72	-0.91	-0.92
**Glycolysis**	Values derived from the Seahorse bioenergetic analysis	Z-score	-0.93	-1.02	-0.15	-0.63	-0.14	-0.05	1.05	1.88
**Cell Volume**	Calculated based on FSC-A and FSC-W scatters, assuming that cells are spheres	Z-score	0.18	0.39	2.09	-0.67	0.23	-0.93	-0.84	-0.45

Supplementary Figure 4. Functional Mitochondrial Characterization and Bioenergetic Profiling of CRC Cell Lines. (**A**) Representative histograms and their quantification demonstrating MMP of CRC cells. Conversion of JC-1 monomers (green fluorescence) into JC-1 J-aggregates (red fluorescence) depends on the MMP. Analysis was performed using flow cytometry and 3 μM JC-1 dye. Values are means ± SEM, n = 3-4. (**B-E**) Bioenergetic analysis of CRC cells with the usage of Seahorse analyser upon subsequent injections of oligomycin, FCCP, rotenone+antimycin A, 2-DG. Values are means ± SEM, n = 3. (**B-C**) Parameters related to oxygen consumption represented by OCR values. (**D-E**) Parameters related to extracellular acidification represented by ECAR values. (**F**) Table illustrating calculation of key parameters used in the study (subsequently used for correlation analysis in figures 5, S12 and S13).

Fig. S5

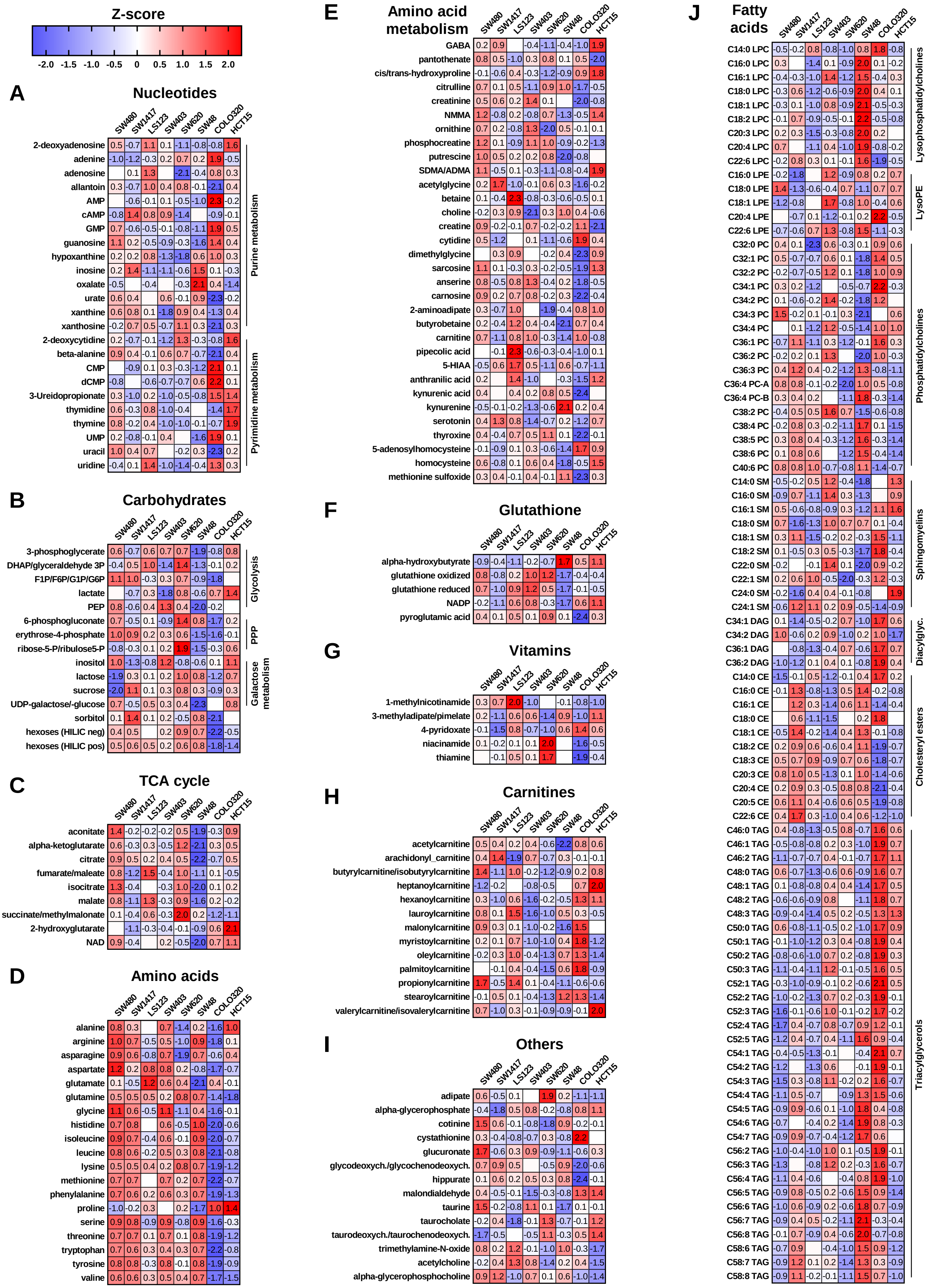
Z-score
-2.0 -1.5 -1.0 -0.5 0 0.5 1.0 1.5 2.0
SW480 SW1417 LS123 SW403 SW620 SW48 COLO320 HCT15
A
Nucleotides
2-deoxyadenosine
adenine
adenosine
allantoin
AMP
cAMP
GMP
guanosine
hypoxanthine
inosine
oxalate
urate
xanthine
xanthosine
2-deoxycytidine
beta-alanine
CMP
dCMP
3-Ureidopropionate
thymidine
thymine
UMP
uracil
uridine
Purine metabolism
Pyrimidine metabolism
B
Carbohydrates
3-phosphoglycerate
DHAP/glyceraldehyde 3P
F1P/F6P/G1P/G6P
lactate
PEP
6-phosphogluconate
erythrose-4-phosphate
ribose-5-P/ribulose5-P
inositol
lactose
sucrose
UDP-galactose/-glucose
sorbitol
hexoses (HILIC neg)
hexoses (HILIC pos)
Glycolysis
PPP
Galactose metabolism
C
TCA cycle
aconitate
alpha-ketoglutarate
citrate
fumarate/maleate
isocitrate
malate
succinate/methylmalonate
2-hydroxyglutarate
NAD
D
Amino acids
alanine
arginine
asparagine
aspartate
glutamate
glutamine
glycine
histidine
isoleucine
leucine
lysine
methionine
phenylalanine
proline
serine
threonine
tryptophan
tyrosine
valine
E
Amino acid metabolism
GABA
pantothenate
cis/trans-hydroxyproline
citrulline
creatinine
NMMA
ornithine
phosphocreatine
putrescine
SDMA/ADMA
acetylglycine
betaine
choline
creatine
cytidine
dimethylglycine
sarcosine
anserine
carnosine
2-aminoadipate
butyrobetaine
carnitine
pipecolic acid
5-HIAA
anthranilic acid
kynurenic acid
kynurenine
serotonin
thyroxine
5-adenosylhomocysteine
homocysteine
methionine sulfoxide
F
Glutathione
alpha-hydroxybutyrate
glutathione oxidized
glutathione reduced
NADP
pyroglutamic acid
G
Vitamins
1-methylnicotinamide
3-methyladipate/pimelate
4-pyridoxate
niacinamide
thiamine
H
Carnitines
acetylcarnitine
arachidonyl_carnitine
butyrylcarnitine/isobutyrylcarnitine
heptanoylcarnitine
hexanoylcarnitine
lauroylcarnitine
malonylcarnitine
myristoylcarnitine
oleylcarnitine
palmitoylcarnitine
propionylcarnitine
stearoylcarnitine
valerylcarnitine/isovalerylcarnitine
I
Others
adipate
alpha-glycerophosphate
cotinine
cystathionine
glucuronate
glycodeoxych./glycochenodeoxych.
hippurate
malondialdehyde
taurine
taurocholate
taurodeoxych./taurochenodeoxych.
trimethylamine-N-oxide
acetylcholine
alpha-glycerophosphocholine
J
Fatty acids
C14:0 LPC
C16:0 LPC
C16:1 LPC
C18:0 LPC
C18:1 LPC
C18:2 LPC
C20:3 LPC
C20:4 LPC
C22:6 LPC
C16:0 LPE
C18:0 LPE
C18:1 LPE
C20:4 LPE
C22:6 LPE
C32:0 PC
C32:1 PC
C32:2 PC
C34:1 PC
C34:2 PC
C34:3 PC
C34:4 PC
C36:1 PC
C36:2 PC
C36:3 PC
C36:4 PC-A
C36:4 PC-B
C38:2 PC
C38:4 PC
C38:5 PC
C38:6 PC
C40:6 PC
C14:0 SM
C16:0 SM
C16:1 SM
C18:0 SM
C18:1 SM
C18:2 SM
C22:0 SM
C22:1 SM
C24:0 SM
C24:1 SM
C34:1 DAG
C34:2 DAG
C36:1 DAG
C36:2 DAG
C14:0 CE
C16:0 CE
C16:1 CE
C18:0 CE
C18:1 CE
C18:2 CE
C18:3 CE
C20:3 CE
C20:4 CE
C20:5 CE
C22:6 CE
C46:0 TAG
C46:1 TAG
C46:2 TAG
C48:0 TAG
C48:1 TAG
C48:2 TAG
C48:3 TAG
C50:0 TAG
C50:1 TAG
C50:2 TAG
C50:3 TAG
C52:1 TAG
C52:2 TAG
C52:3 TAG
C52:4 TAG
C52:5 TAG
C54:1 TAG
C54:2 TAG
C54:3 TAG
C54:4 TAG
C54:5 TAG
C54:6 TAG
C54:7 TAG
C56:2 TAG
C56:3 TAG
C56:4 TAG
C56:5 TAG
C56:6 TAG
C56:7 TAG
C56:8 TAG
C58:6 TAG
C58:7 TAG
C58:8 TAG
Lysophosphatidylcholines
LysoPE
Phosphatidylcholines
Sphingomyelins
Diacylglyc.
Cholesteryl esters
Triacylglycerols

Supplementary Figure 5. Categorization and Distribution of Metabolite Abundances in CRC Cell Lines. (**A-J**) The values characterizing the abundance of metabolites in CRC cell lines were obtained from the DepMap portal and CCLE project. After data extraction, the values were Z-normalized and manually grouped by metabolite categories.

Fig.S6

Compound:	Primary Target:	Concentration:
**Rotenone**	Complex I	0.2 μM
**Antimycin A**	Complex III	0.2 μM
**Oligomycin**	Complex V	0.1 μM
**CCCP**	Mitochondrial uncoupler	50 nM
**UK-5099**	Pyruvate	20 μM
**Etomoxir**	Fatty acid oxidation	5 μM
**BPTES**	Glutaminolysis	0.5 μM
**Dimethyl fumarate**	TCA cycle	10 μM
**Mdivi-1**	Fusion/fission dynamics	20 μM
**Cyclosporine A (CsA)**	MPTP	200 nM
**2-Deoxy-D-Glucose (2-DG)**	Glycolysis	200 μM
**AZD3965**	Inhibits monocarboxylate transporters	10 nM
**EIPA (Ethylisopropylamiloride)**	Inhibits sodium-hydrogen exchangers	1 μM
**Topiramate**	Inhibits carbonic anhydrases	1 μM
**Concanamycin A**	Glycolysis	2 nM
**Irinotecan**	Blocks topoisomerase-1	2 μM
**Oxaliplatin**	Crosslinks DNA	2 μM
**5-Fluorouracil (5-FU)**	Inhibitor of thymidylate synthase	5 μM

Mechanism of action of selected compounds targeting various metabolic and adjacent homeostatic pathways

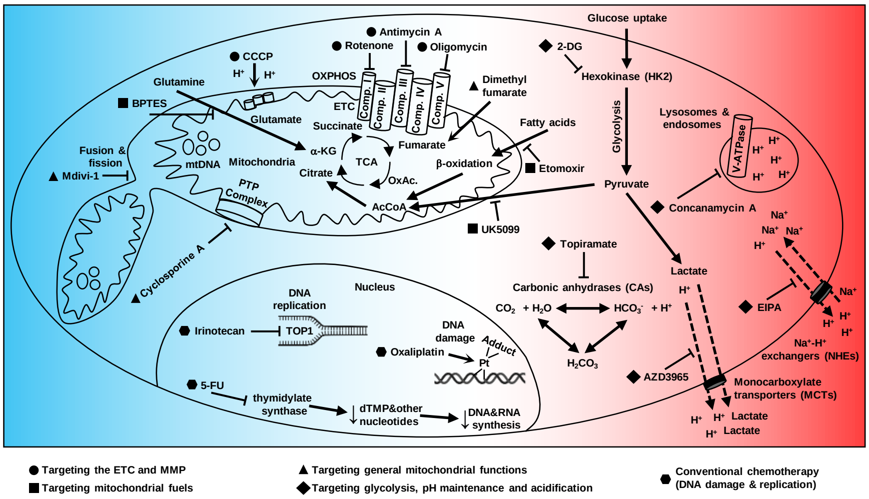

Supplementary Figure 6. Compounds Targeting Key Metabolic and Homeostatic Processes. List of compounds selected for the study, detailing their concentrations and targets. The schematic illustration depicts their mechanisms of action. The compounds were divided into 5 groups owing to some similarities or common effects in their mechanism of action.

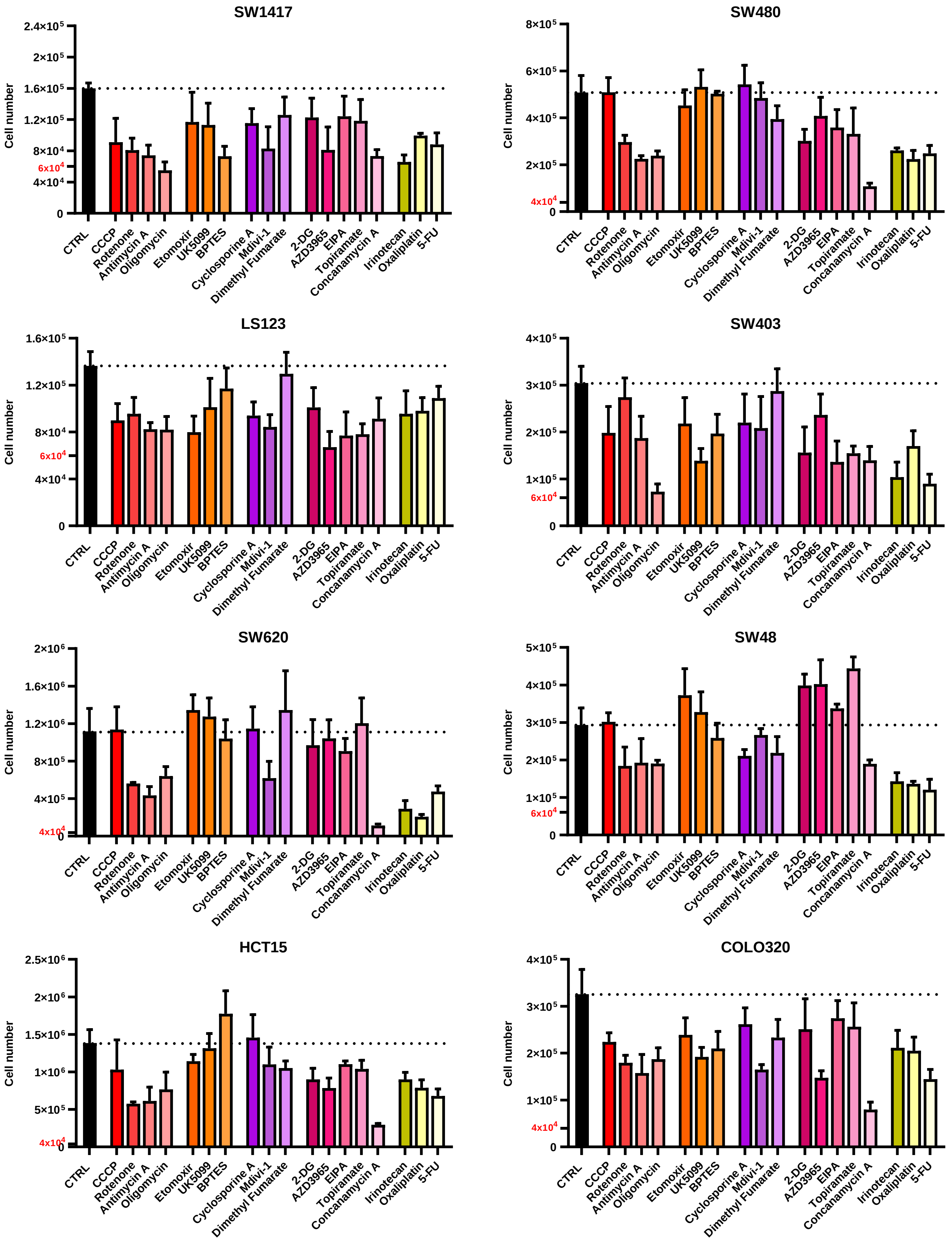

Supplementary Figure 7. Changes in Cell Number Following Pharmacological Impairment of Metabolic and Homeostatic Processes in CRC Cells. CRC cells were seeded one day prior to the beginning of the experiment and then treated with selected compounds for 72 hours. Subsequently, analysis of cell number was carried out. The changes in cell number were quantified using flow cytometry. Only events corresponding to normal cell size were considered. Values are means ± SEM, n = 3-4.

Fig.S8

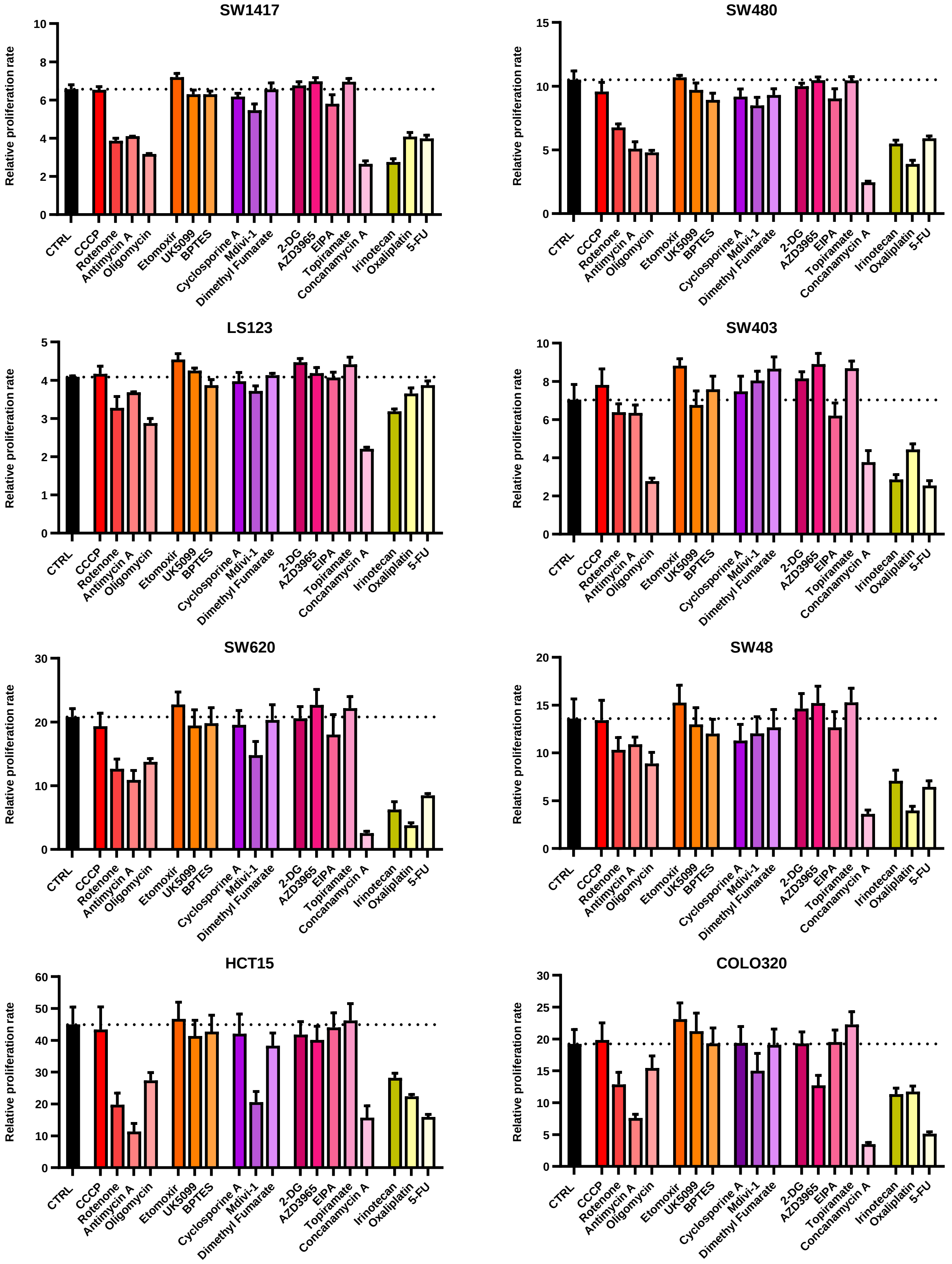

Supplementary Figure 8. Changes in Proliferation Rate Following Pharmacological Impairment of Metabolic and Homeostatic Processes in CRC Cells. CRC cells were prestained with 3 μM of CellTrace Violet dye and seeded one day prior to the start of the experiment. On day 1 (D1), they were treated with selected compounds for 72 hours. Subsequently, the proliferation rate was analysed on day 4 (D4). The proliferation rate was calculated using the D1/D4 ratio. Values are means ± SEM, n = 3-4.

Fig.S9

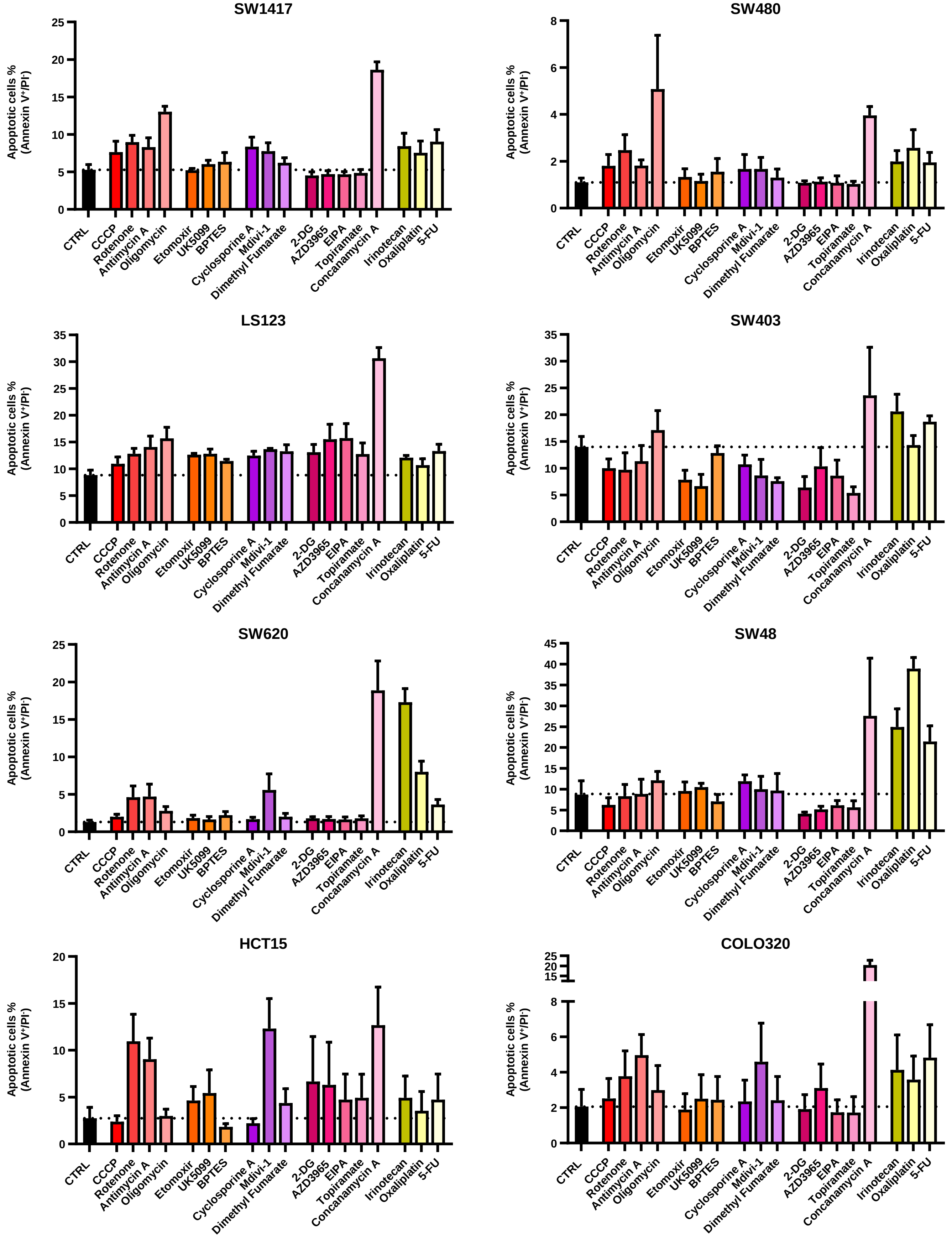

Supplementary Figure 9. Changes in Apoptosis Rate Following Pharmacological Impairment of Metabolic and Homeostatic Processes in CRC Cells. CRC cells were seeded one day prior to the beginning of the experiment and then treated with selected compounds for 72 hours. Subsequently, analysis of apoptosis levels was carried out. The changes in apoptosis levels were quantified using flow cytometry along with annexin V staining. Values are means ± SEM, n = 3-4.

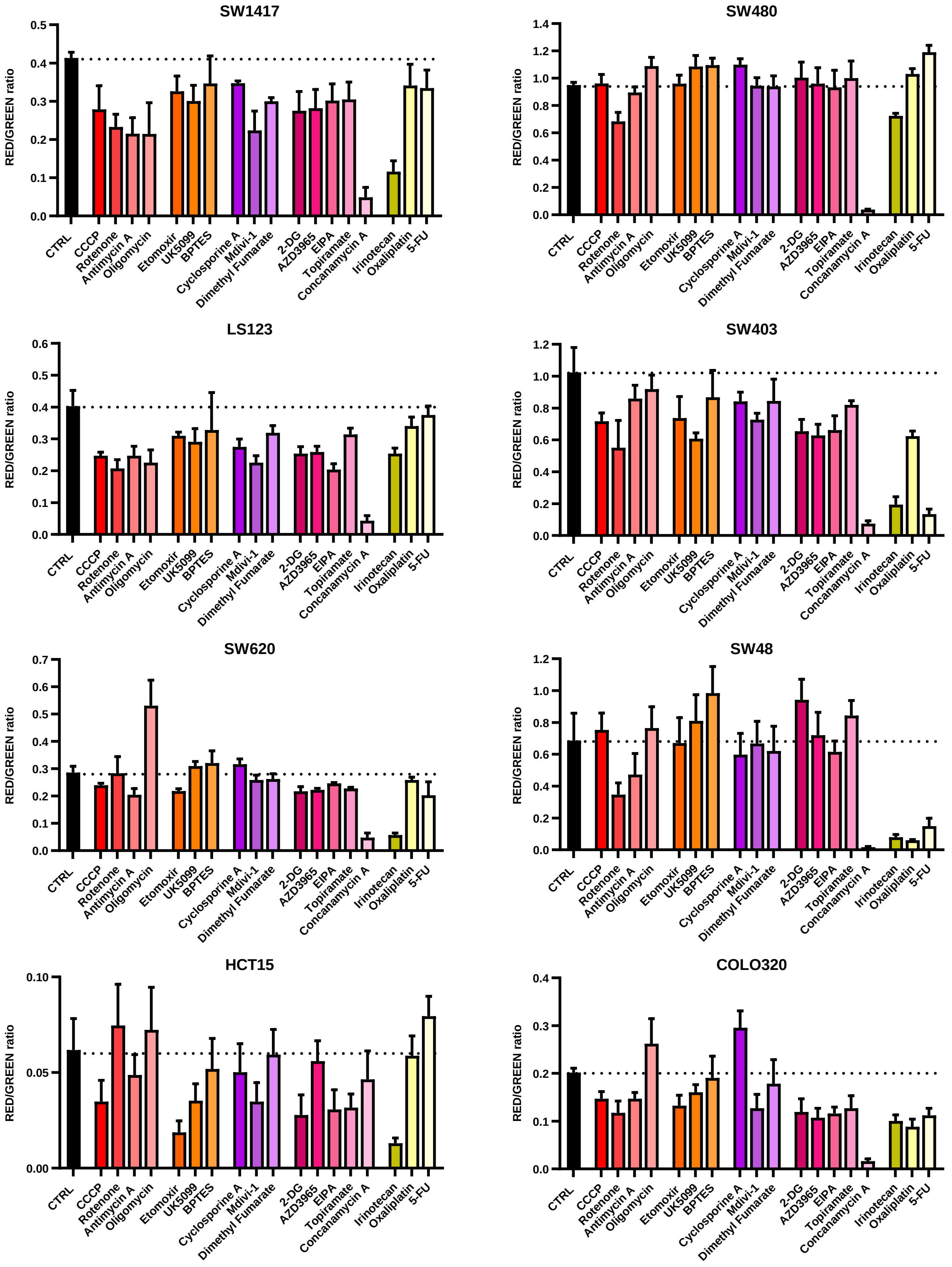

Supplementary Figure 10. Changes in MMP Following Pharmacological Impairment of Metabolic and Homeostatic Processes in CRC Cells.. CRC cells were seeded one day prior to the beginning of the experiment and then treated with selected drugs for 72 hours. Subsequently, analysis of MMP was carried out. The changes in MMP levels were quantified using flow cytometry along with 3 μM JC-1 staining. Values are means ± SEM, n = 3-4.

Fig.S11

Calculation of Pearson correlation matrices

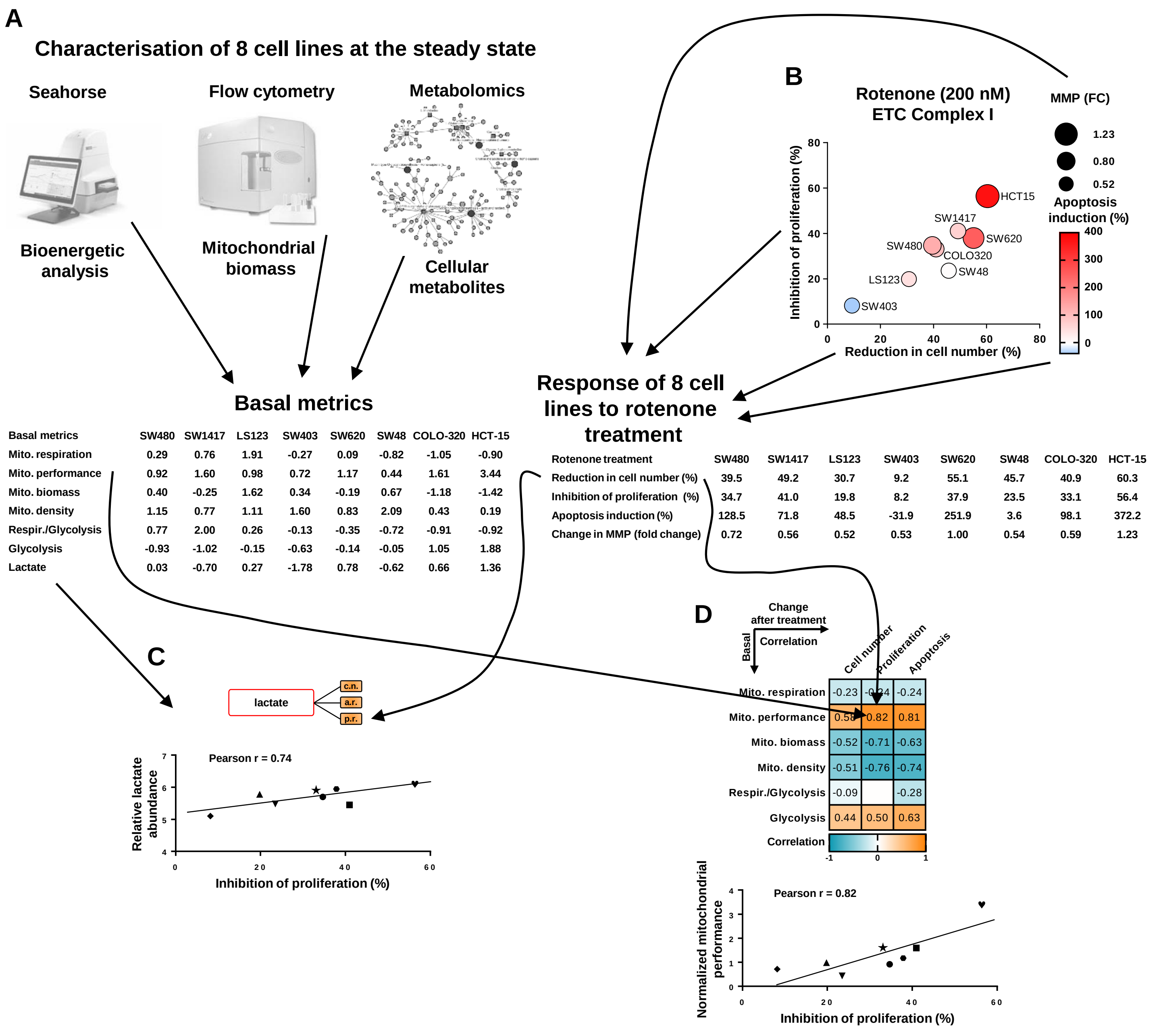

Basal metrics	SW480	SW1417	LS123	SW403	SW620	SW48	COLO-320	HCT-15
Mito. respiration	0.29	0.76	1.91	-0.27	0.09	-0.82	-1.05	-0.90
Mito. performance	0.92	1.60	0.98	0.72	1.17	0.44	1.61	3.44
Mito. biomass	0.40	-0.25	1.62	0.34	-0.19	0.67	-1.18	-1.42
Mito. density	1.15	0.77	1.11	1.60	0.83	2.09	0.43	0.19
Respir./Glycolysis	0.77	2.00	0.26	-0.13	-0.35	-0.72	-0.91	-0.92
Glycolysis	-0.93	-1.02	-0.15	-0.63	-0.14	-0.05	1.05	1.88
Lactate	0.03	-0.70	0.27	-1.78	0.78	-0.62	0.66	1.36

Rotenone treatment	SW480	SW1417	LS123	SW403	SW620	SW48	COLO-320	HCT-15
Reduction in cell number (%)	39.5	49.2	30.7	9.2	55.1	45.7	40.9	60.3
Inhibition of proliferation (%)	34.7	41.0	19.8	8.2	37.9	23.5	33.1	56.4
Apoptosis induction (%)	128.5	71.8	48.5	-31.9	251.9	3.6	98.1	372.2
Change in MMP (fold change)	0.72	0.56	0.52	0.53	1.00	0.54	0.59	1.23

	Cell number	Proliferation	Apoptosis
Mito. respiration	-0.23	-0.24	-0.24
Mito. performance	0.58	0.82	0.81
Mito. biomass	-0.52	-0.71	-0.63
Mito. density	-0.51	-0.76	-0.74
Respir./Glycolysis	-0.09		-0.28
Glycolysis	0.44	0.50	0.63

Supplementary Figure 11. Calculation of Pearson Correlation Matrices Linking Drug Response Parameters and Steady-State Characteristics. CRC cells were either analysed at the steady state or following the 72 hours treatment with rotenone. (**A**) Bioenergetic characteristics, mitochondrial parameters and the levels of metabolites were obtained at the steady state using seahorse assay, flow cytometry and CCLE database, respectively. (**B**) Following the 72-hour treatment with rotenone, analysis of cell number, apoptosis levels, proliferation rate and MMP was carried out (Figures S7-S10). The obtained data was quantified and represented as fold changes or percentages over the control condition and visualised as bubble plots. These bubble plots illustrate the mean changes in cell number (x-axis, %), mean changes in proliferation rate (y-axis, %), mean changes in apoptosis (colour, %) and mean changes in MMP (size, fold change). The changes in cell number, proliferation rate, apoptosis levels and MMP were quantified using flow cytometry along with CellTrace Violet, annexin V, and JC-1 stainings, respectively. Following the evaluation of steady state-characteristics and drug response changes induced by rotenone treatment, we assessed the linkage between these parameters. (**C**) **Top:** Scheme depicting associations between the abundance of lactate and rotenone-induced drug-response parameters in CRC cells. Each line between lactate levels and a response parameter such as change in cell number (c.n.), apoptosis rate (a.r.) and proliferation rate (p.r) depicts a strong correlation (<-0.6, blue-filled box; > 0.6, orange-filled box). The colour of the frame depicts if metabolites accumulate in cells with specific bioenergetic profiles. The legends are explained in detail in Figure S12A.
(**C**) **Bottom:** Illustration of strong positive correlations between the levels of lactate and inhibition of proliferation induced by rotenone. (**D**) **Top:** Correlation matrix depicting the relationships between various mitochondrial and bioenergetic characteristics measured at the steady level and the changes in cell number, proliferation and apoptosis rates induced by rotenone. (**D**) **Bottom:** Illustration of a strong positive correlation between normalized mitochondrial performance and inhibition of proliferation induced by rotenone.

Fig.S12

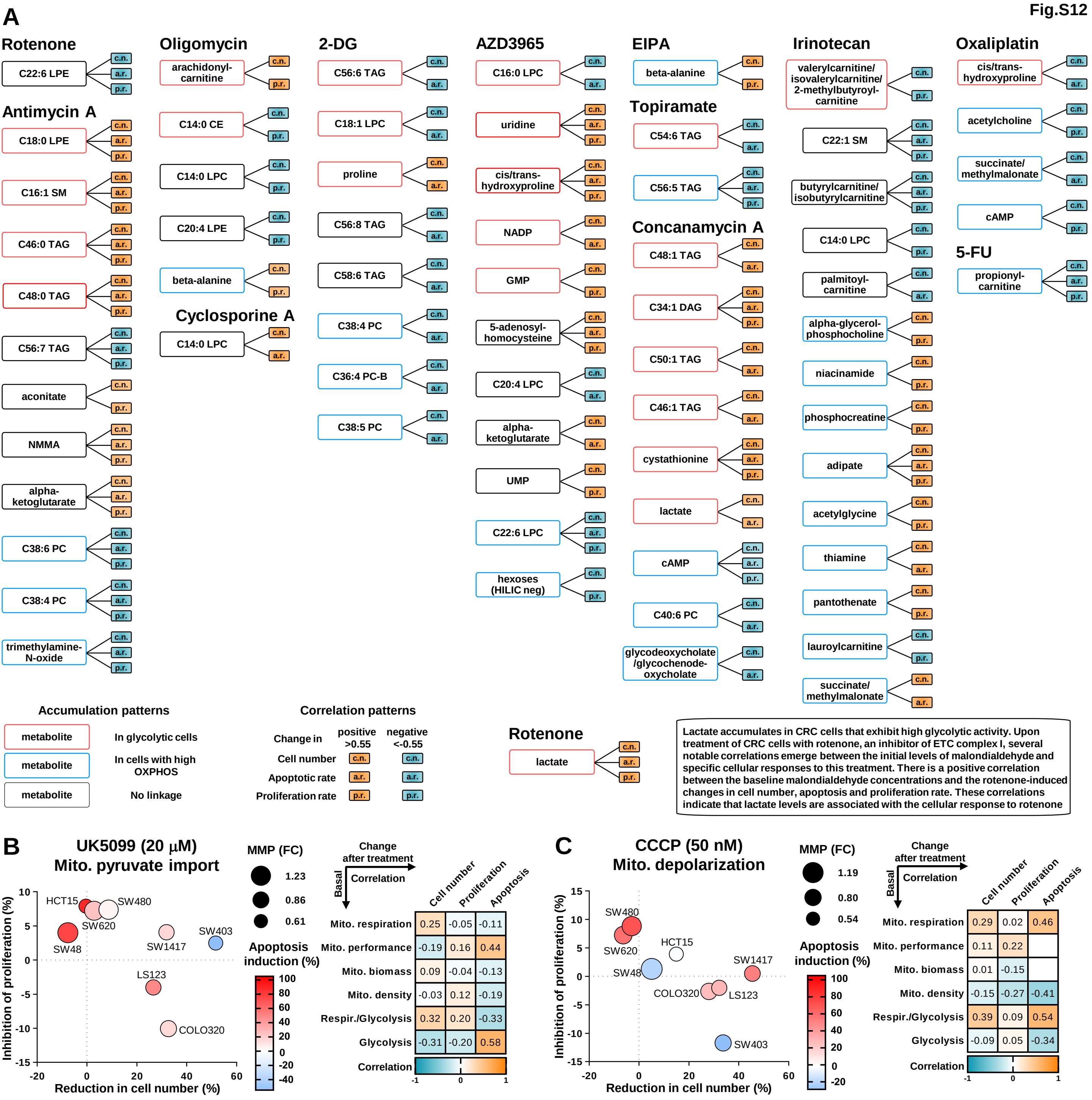

Supplementary Figure 12. Correlating Drug Efficacy with Bioenergetics, Mitochondrial Parameters and Metabolite Levels in CRC Cells (extension, part II). (**A-C**) CRC cells were seeded one day prior to the beginning of the experiment and then treated with selected drugs in indicated concentrations for 72 hours. Subsequently, analysis of cell number, apoptosis levels, proliferation rate and MMP was carried out (Figures S7-S10). The obtained data was quantified and represented as fold changes or percentages over the control condition. (**A**) The correlations between the abundance of metabolites and the drug response triggered by each specific compound in the panel of CRC cells (extension of data present in Figures 5 and S13). Each line between a metabolite and a response parameter depicts a strong correlation (<-0.6, blue-filled box; > 0.6, orange-filled box). Only metabolites displaying at least two simultaneous strong correlations are depicted. The drug response is represented by three parameters such as changes in cell number, apoptosis level and proliferation rate. The colour of the frames depicts whether metabolites are accumulated in cells with specific bioenergetic profiles. (**B-C**) **Left side:** Bubble plots illustrate the mean changes in cell number (x-axis, %), mean changes in proliferation rate (y-axis, %), mean changes in apoptosis (colour, %) and mean changes in MMP (size, fold change). The changes in cell number, proliferation rate, apoptosis levels and MMP were quantified using flow cytometry along with CellTrace Violet, annexin V, and JC-1 stainings, respectively. (**B-C**) **Right side:** Correlation matrices depict the relationships between various mitochondrial and bioenergetic characteristics measured at the steady level and the drug responses induced by treatments with selected compounds (see Figure S11). The drug response is represented by changes in cell number, proliferation rate and apoptosis.

Fig.S13

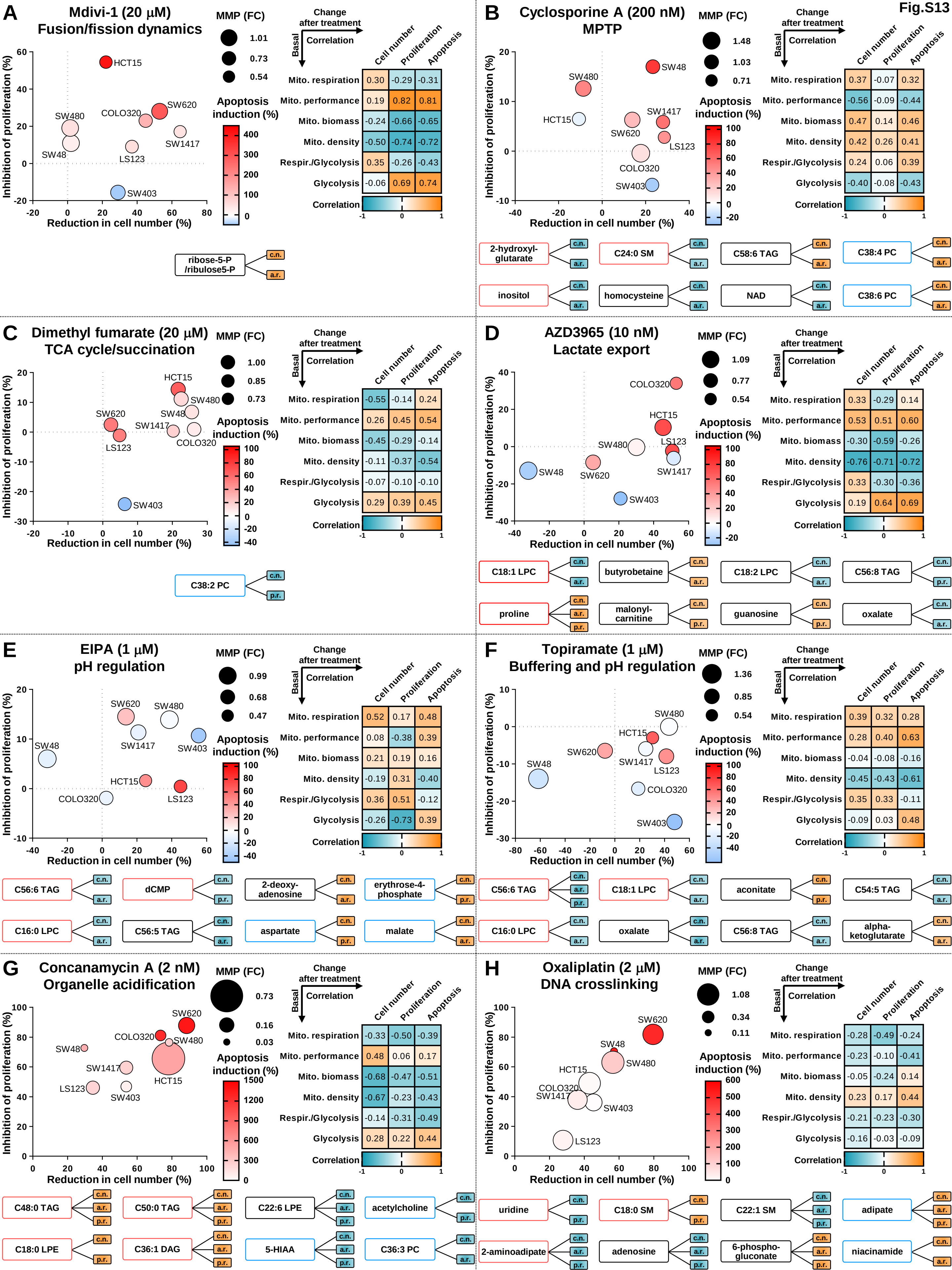

A
Mdivi-1 (20 µM)
Fusion/fission dynamics
MMP (FC)
1.01
0.73
0.54
Apoptosis induction (%)
400
300
200
100
0
Inhibition of proliferation (%)
Reduction in cell number (%)
HCT15
SW480
COLO320
SW620
SW48
LS123
SW1417
SW403
Change after treatment
Basal
Correlation
Cell number
Proliferation
Apoptosis
Mito. respiration 0.30 -0.29 -0.31
Mito. performance 0.19 0.82 0.81
Mito. biomass -0.24 -0.66 -0.65
Mito. density -0.50 -0.74 -0.72
Respir./Glycolysis 0.35 -0.26 -0.43
Glycolysis -0.06 0.69 0.74
Correlation -1 0 1
ribose-5-P /ribulose5-P
c.n.
a.r.
B
Cyclosporine A (200 nM)
MPTP
MMP (FC)
1.48
1.03
0.71
Apoptosis induction (%)
100
80
60
40
20
0
-20
Inhibition of proliferation (%)
Reduction in cell number (%)
SW480
SW48
HCT15
SW1417
SW620
LS123
COLO320
SW403
Mito. respiration 0.37 -0.07 0.32
Mito. performance -0.56 -0.09 -0.44
Mito. biomass 0.47 0.14 0.46
Mito. density 0.42 0.26 0.41
Respir./Glycolysis 0.24 0.06 0.39
Glycolysis -0.40 -0.08 -0.43
2-hydroxyl-glutarate
C24:0 SM
C58:6 TAG
C38:4 PC
inositol
homocysteine
NAD
C38:6 PC
C
Dimethyl fumarate (20 µM)
TCA cycle/succination
MMP (FC)
1.00
0.85
0.73
Apoptosis induction (%)
100
80
60
40
20
0
-20
-40
HCT15
SW480
SW48
SW620
SW1417
LS123
COLO320
SW403
Mito. respiration -0.55 -0.14 0.24
Mito. performance 0.26 0.45 0.54
Mito. biomass -0.45 -0.29 -0.14
Mito. density -0.11 -0.37 -0.54
Respir./Glycolysis -0.07 -0.10 -0.10
Glycolysis 0.29 0.39 0.45
C38:2 PC
c.n.
p.r.
D
AZD3965 (10 nM)
Lactate export
MMP (FC)
1.09
0.77
0.54
Apoptosis induction (%)
100
80
60
40
20
0
-20
COLO320
HCT15
SW480
LS123
SW1417
SW48
SW620
SW403
Mito. respiration 0.33 -0.29 0.14
Mito. performance 0.53 0.51 0.60
Mito. biomass -0.30 -0.59 -0.26
Mito. density -0.76 -0.71 -0.72
Respir./Glycolysis 0.33 -0.30 -0.36
Glycolysis 0.19 0.64 0.69
C18:1 LPC
butyrobetaine
C18:2 LPC
C56:8 TAG
proline
malonyl-carnitine
guanosine
oxalate
E
EIPA (1 µM)
pH regulation
MMP (FC)
0.99
0.68
0.47
Apoptosis induction (%)
100
80
60
40
20
0
-20
-40
SW620
SW480
SW48
SW1417
SW403
HCT15
LS123
COLO320
Mito. respiration 0.52 0.17 0.48
Mito. performance 0.08 -0.38 0.39
Mito. biomass 0.21 0.19 0.16
Mito. density -0.19 0.31 -0.40
Respir./Glycolysis 0.36 0.51 -0.12
Glycolysis -0.26 -0.73 0.39
C56:6 TAG
dCMP
2-deoxy-adenosine
erythrose-4-phosphate
C16:0 LPC
C56:5 TAG
aspartate
malate
F
Topiramate (1 µM)
Buffering and pH regulation
MMP (FC)
1.36
0.85
0.54
Apoptosis induction (%)
100
80
60
40
20
0
-20
-40
SW480
HCT15
SW620
SW1417
LS123
SW48
COLO320
SW403
Mito. respiration 0.39 0.32 0.28
Mito. performance 0.28 0.40 0.63
Mito. biomass -0.04 -0.08 -0.16
Mito. density -0.45 -0.43 -0.61
Respir./Glycolysis 0.35 0.33 -0.11
Glycolysis -0.09 0.03 0.48
C56:6 TAG
C18:1 LPC
aconitate
C54:5 TAG
C16:0 LPC
oxalate
C56:8 TAG
alpha-ketoglutarate
G
Concanamycin A (2 nM)
Organelle acidification
MMP (FC)
0.73
0.16
0.03
Apoptosis induction (%)
1500
1200
900
600
300
0
SW620
COLO320
SW480
SW48
SW1417
HCT15
LS123
SW403
Mito. respiration -0.33 -0.50 -0.39
Mito. performance 0.48 0.06 0.17
Mito. biomass -0.68 -0.47 -0.51
Mito. density -0.67 -0.23 -0.43
Respir./Glycolysis -0.14 -0.31 -0.49
Glycolysis 0.28 0.22 0.44
C48:0 TAG
C50:0 TAG
C22:6 LPE
acetylcholine
C18:0 LPE
C36:1 DAG
5-HIAA
C36:3 PC
H
Oxaliplatin (2 µM)
DNA crosslinking
MMP (FC)
1.08
0.34
0.11
Apoptosis induction (%)
600
500
400
300
200
100
0
SW620
SW48
SW480
HCT15
COLO320
SW1417
SW403
LS123
Mito. respiration -0.28 -0.49 -0.24
Mito. performance -0.23 -0.10 -0.41
Mito. biomass -0.05 -0.24 0.14
Mito. density 0.23 0.17 0.44
Respir./Glycolysis -0.21 -0.23 -0.30
Glycolysis -0.16 -0.03 -0.09
uridine
C18:0 SM
C22:1 SM
adipate
2-aminoadipate
adenosine
6-phospho-gluconate
niacinamide

Supplementary Figure 13. Correlating Drug Efficacy with Bioenergetics, Mitochondrial Parameters and Metabolite Levels in CRC Cells (extension, part III). (**A-H**) CRC cells were seeded one day prior to the beginning of the experiment and then treated with selected drugs in indicated concentrations for 72 h. Subsequently, analysis of cell number, apoptosis levels, proliferation rate and MMP was carried out (Figures S7-S10). The obtained data was quantified and represented as fold changes (FC) or percentages over the control condition. (**A-H**) **Left side:** Bubble plots illustrate the mean changes in cell number (x-axis, %), mean changes in proliferation rate (y-axis, %), mean changes in apoptosis (colour, %) and mean changes in MMP (size, fold change). The changes in cell number, proliferation rate, apoptosis levels and MMP were quantified using flow cytometry along with CellTrace Violet, annexin V, and JC-1 stainings, respectively. (**A-H**) **Right side:** Correlation matrices depict the relationships between various mitochondrial and bioenergetic characteristics measured at the steady level and the drug responses induced by treatments with selected compounds (see Figure S11). The drug response is represented by changes in cell number, proliferation rate and apoptosis. (**A-H**) **bottom:** The correlations between the abundance of metabolites and the drug response parameters triggered by each specific compound in the panel of CRC cells (Details continue in Figure S12). Each line between a metabolite and a response parameter depicts a strong correlation (<-0.6, blue-filled box; > 0.6, orange-filled box). Only metabolites displaying at least two simultaneous strong correlations are depicted. The drug response is represented by the three parameters change in cell number (c.n.), apoptosis rate (a.r.) and proliferation rate (p.r). The colour of the frames depicts whether metabolites are accumulated in cells with specific bioenergetic profiles. The legends are explained in detail in Figure S12A.

Fig.S14

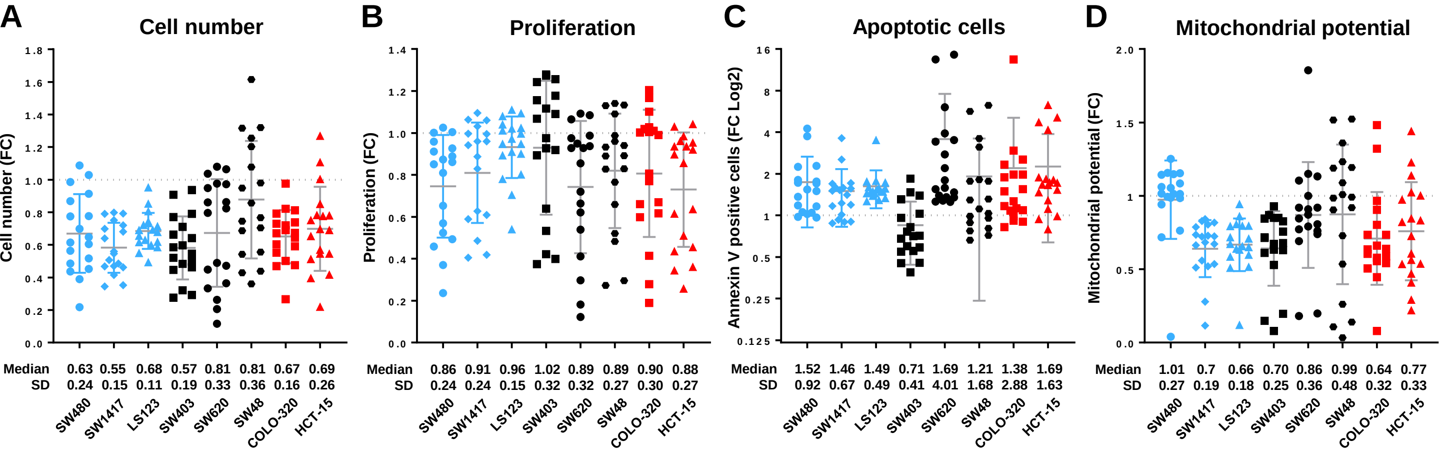

Supplementary Figure 14. Overview of Cellular Responses to Metabolic and Homeostatic Treatments in CRC Cells. (**A-D**) Summary plots depicting the changes in cell number, proliferation rate, apoptosis level and mitochondrial potential upon treatment of cells with 18 different compounds. Bioenergetic clusters are colour-coded (blue: high OXPHOS, black: intermediate, red: high glycolysis). The graphs represent a summary and contain data derived from other figures. Each dot represents the mean effect of one compound with a line characterising the mean effect of 18 compounds, error bars are ± SD.

Fig. S15

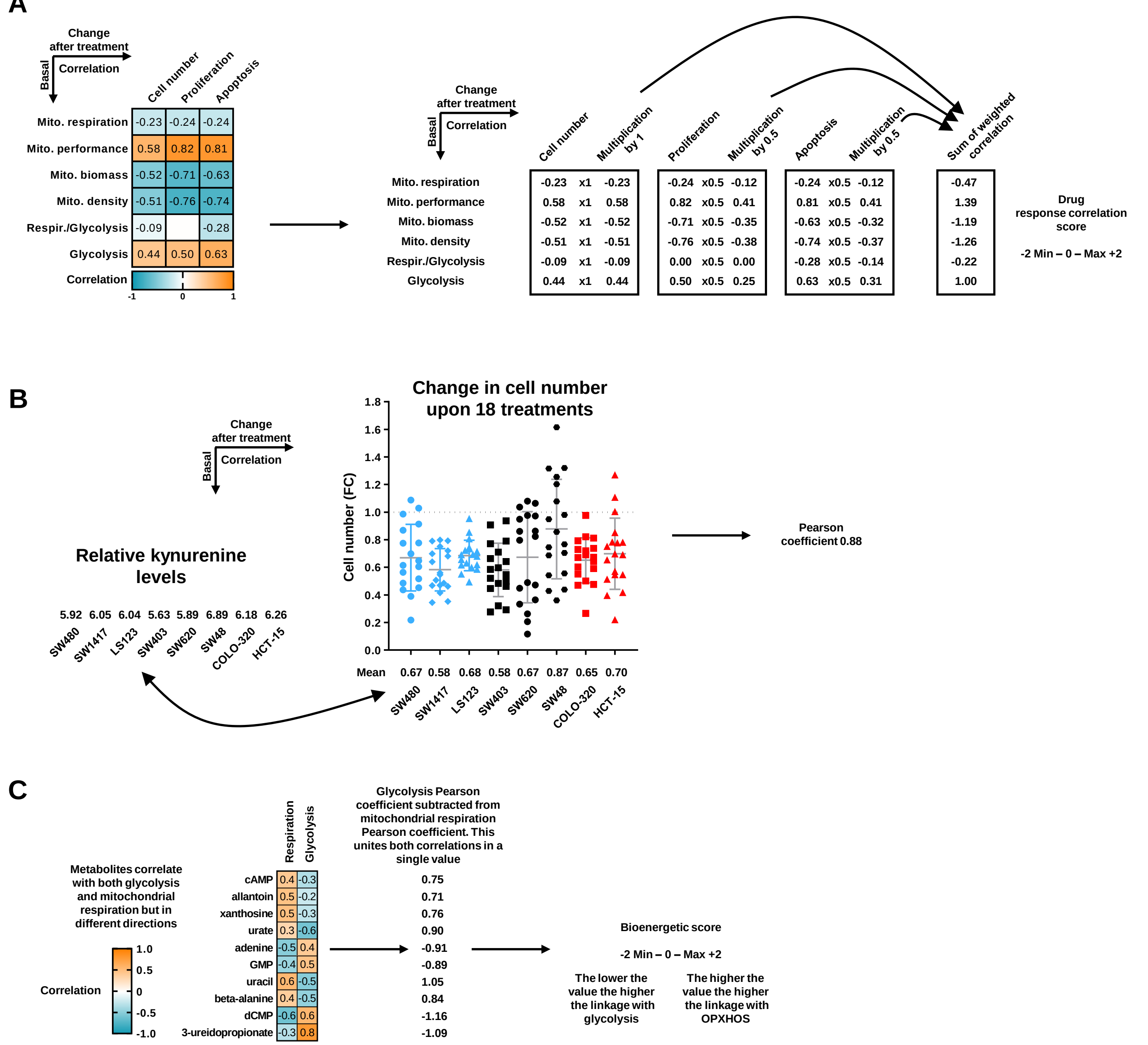

Supplementary Figure 15. Calculation of Bioenergetic and Drug Response Correlation Scores. (**A**) To better understand the relationship between mitochondrial/bioenergetic characteristics and cell responses to drug treatments, we introduced a "drug response correlation score." This score integrates the treatment-induced changes in cell number, proliferation rate, and apoptosis levels, with weighted values to reflect their significance. Specifically, the change in cell number is given a weight of 1.0, acknowledging its primary importance, while changes in proliferation and apoptosis are each weighted at 0.5. Thus, the score is calculated as the sum of these weighted correlation coefficients, represented as 1.0 x P_{CCvsBM} (treatment-induced change in cell number vs basal metric) + 0.5 x P_{PCvsBM} (treatment-induced change in proliferation rate vs basal metric) + 0.5 x P_{ACvsBM} (treatment-induced change in apoptosis levels vs basal metric), where P represents Pearson correlation coefficients. This provides a broader assessment of drug effects, ranging from -2 to 2, thereby offering a wider range than the conventional Pearson coefficient scale of -1 to 1. (**B**) Basal levels of metabolites in CRC cells were compared with the average reduction in cell number upon 18 treatments by examining correlation values between these two parameters. Each dot represents the mean reduction in cell number induced by one out of 18 treatments. (**C**) Considering that abundance of any metabolite can be simultaneously linked with both mitochondrial respiration and glycolysis levels, we aimed to utilize this dual association to enhance the resolution of our analysis. To achieve this, we introduced a "metabolite bioenergetic score" that integrates a metabolite's correlation with glycolysis and mitochondrial respiration into a single value. This bioenergetic score is calculated by subtracting the Pearson correlation coefficient for glycolysis from the Pearson correlation coefficient for mitochondrial respiration. A higher score indicates that a metabolite is positively associated with OXPHOS and negatively with glycolysis, whereas a lower score suggests that a metabolite is negatively associated with OXPHOS and positively with glycolysis. This scoring system allows for a broader assessment of metabolite association with bioenergetic processes, providing a range from -2 to 2, compared to the conventional Pearson coefficient range of -1 to 1.

Fig.S16

A

Cell number fold change vs metabolites levels

	CCCP	UK5099	BPTES	CsA	Antimycin A	Concom	Mdivi	Oligomycin	Etomoxir	AZD3965	2-DG	Topiramate	EIPA	Rotenone	fumarate	Irinotecan	Oxaliplatin	5-FU
C38:2 PC	0.4	0.7	0.5	0.4	-0.2	0.1	0.5	0.8	0.6	0.3	0.7	0.6	0.7	-0.6	-0.8	0.4		
aconitate	-0.3		-0.3	-0.7	0.6	0.8		0.3	0.4	0.5	0.7	0.7	0.6	0.2	-0.2		0.2	-0.2
isocitrate	-0.3		-0.2	-0.6	0.6	0.8	0.2	0.2	0.4	0.5	0.7	0.6	0.6	0.1	-0.3		0.2	-0.2
alpha-ketoglutarate	-0.2		-0.2	-0.5	0.6	0.8	0.4	0.2	0.4	0.6	0.6	0.6	0.5	0.2	-0.4	-0.1	0.1	-0.3
citrate	0.1	0.3		-0.4	0.4	0.6	0.3	0.6	0.7	0.6	0.8	0.8	0.8		-0.4	0.1		-0.3
putrescine		0.2	0.1	-0.3	0.5	0.6	0.3	0.6	0.5	0.5	0.7	0.7	0.8		-0.5	0.3	0.1	-0.2
C34:3 PC	-0.1	0.1	-0.2	-0.6	0.5	0.7	0.1	0.3	0.6	0.6	0.8	0.8	0.7		-0.2	-0.1		-0.3
UDP-galactose/UDP-glucose		0.3	-0.2	-0.5	0.3	0.6	0.2	0.3	0.7	0.7	0.9	0.8	0.8	-0.1	-0.4	-0.2	-0.1	-0.3
homocysteine	-0.1	0.1	-0.5	-0.7	0.4	0.6		0.3	0.5	0.4	0.8	0.7	0.7		-0.4	-0.1	0.1	-0.1
3-phosphoglycerate	-0.2	0.1	-0.3	-0.5	0.3	0.5	0.1	0.4	0.5	0.4	0.8	0.7	0.8	-0.2	-0.6	0.1	0.1	-0.2
PEP		0.5		-0.3	0.1	0.5	0.1	0.6	0.6	0.4	0.9	0.8	0.9	-0.5	-0.6	0.1		
glutathione reduced		0.5	0.1	-0.1	-0.1	0.3	0.1	0.4	0.6	0.3	0.8	0.7	0.8	-0.6	-0.7	0.1		-0.1
glutathione oxidized	-0.2	0.3	0.1	-0.2	0.3	0.5	0.1	0.5	0.3	0.2	0.7	0.6	0.7	-0.4	-0.7	0.4	0.4	0.1
taurine	-0.1	0.4	0.2	-0.5	0.3	0.6		0.7	0.5	0.3	0.8	0.7	0.7	-0.4	-0.1	0.3	0.1	0.2
carnitine	0.1	0.6	0.2		-0.3	0.2		0.1	0.6	0.4	0.7	0.7	0.6	-0.7	-0.3	-0.2	-0.3	-0.2
C36:2 PC	0.5	0.9	0.4	0.1	-0.1	0.3	0.5	0.6	0.7	0.7	0.9	0.8	0.7	-0.5	-0.4		-0.3	
C22:0 SM	0.4	0.8	0.4	0.1	-0.1	0.4	0.5	0.6	0.7	0.6	0.9	0.7	0.7	-0.5	-0.4		-0.3	0.1
C34:2 PC	0.3	0.7	0.2	-0.1	-0.1	0.4	0.2	0.5	0.6	0.5	0.9	0.7	0.6	-0.5	-0.2	-0.1	-0.2	0.1
C18:2 SM	0.3	0.7	0.3		-0.1	0.4	0.3	0.2	0.6	0.7	0.7	0.6	0.4	-0.4	-0.1	-0.4	-0.4	-0.1
glutamate	0.2	0.5	0.1			0.3	0.4	0.3	0.7	0.7	0.8	0.8	0.8	-0.4	-0.6	-0.2	-0.3	-0.4
acetylcarnitine	0.5	0.6	0.1	-0.3	0.1	0.5	0.4	0.5	0.9	0.9	0.9	0.9	0.7	-0.2		-0.4	-0.5	-0.4
butyrobetaine	0.4	0.5	-0.1	-0.2	-0.1	0.3	0.3	0.2	0.8	0.8	0.8	0.9	0.8	-0.3	-0.3	-0.5	-0.5	-0.5
NADP	0.2	0.4	-0.3	-0.3	-0.1	0.4	0.1	0.1	0.7	0.6	0.8	0.7	0.7	-0.3	-0.3	-0.5	-0.4	-0.2
C14:0 SM	0.4	0.5	-0.2	-0.2	-0.1	0.3	0.3	0.5	0.8	0.7	0.8	0.8	0.8	-0.3	-0.4	-0.3	-0.4	-0.2
glucuronate		0.3	-0.2	-0.5		0.2	-0.5	0.3	0.6	0.3	0.7	0.8	0.8	-0.5	-0.1	-0.1	-0.2	-0.2
propionylcarnitine		0.2		-0.2	-0.1		-0.2	0.1	0.6	0.4	0.5	0.7	0.7	-0.4	-0.3	-0.2	-0.2	-0.6
NAD	0.1	0.3	-0.3	-0.6	0.2	0.6	0.1	0.3	0.7	0.8	0.8	0.8	0.7	-0.1		-0.4	-0.3	-0.3
5-adenosylhomocysteine	0.2	0.4	-0.2	-0.3		0.4	0.1		0.5	0.7	0.6	0.6	0.3	-0.2	0.1	-0.6	-0.4	-0.2
C32:1 PC		0.4		-0.4	0.3	0.7	0.2	0.3	0.4	0.6	0.8	0.6	0.4	-0.1		-0.2	-0.1	0.1
2-deoxyadenosine	0.1		-0.7	-0.5	-0.2		-0.4	-0.1	0.6	0.4	0.4	0.6	0.6	-0.1	-0.1	-0.5	-0.4	-0.5
cis/trans-hydroxyproline	0.2	0.1	-0.6	-0.5	-0.1	0.2	-0.1	-0.3	0.5	0.6	0.4	0.5	0.2	0.1	0.3	-0.8	-0.6	-0.4
lactate	-0.4	-0.5	-0.7	-0.5	0.6	0.6	0.1	-0.6	-0.1	0.3			-0.2	0.7	0.1	-0.5	0.1	-0.4
malondialdehyde	-0.1	-0.3	-0.6	-0.6	0.4	0.5		-0.5	0.1	0.5	0.1	0.2	-0.1	0.6	0.5	-0.7	-0.2	-0.3
2-deoxycytidine	-0.6	-0.7	-0.8	-0.7	0.7	0.5	-0.1	-0.3	-0.3	-0.1	-0.1	-0.1	-0.1	0.7	-0.1		0.5	-0.1
NMMA	-0.5	-0.4	-0.7	-0.8	0.6	0.7	-0.1	-0.1	0.2	0.3	0.4	0.4	0.4	0.4	-0.1	-0.2	0.2	-0.3
C18:0 LPE	-0.6	-0.2	-0.5	-0.8	0.6	0.9	-0.2	-0.1		0.2	0.4	0.3	0.2	0.2	0.1	-0.1	0.4	0.1
hypoxanthine	0.3	-0.1	-0.2		-0.4	-0.4	-0.2	-0.6	0.1	0.3	-0.3		-0.3	0.1	0.7	-0.8	-0.7	-0.6
adenosine	0.6	0.4		0.1	-0.6	-0.4	-0.1	-0.2	0.7	0.6	0.3	0.5	0.3	-0.3	0.3	-0.8	-0.9	-0.7
C22:1 SM	0.6	0.4	0.2	0.2	-0.5	-0.4		-0.2	0.5	0.6	0.1	0.4	0.1	-0.2	0.4	-0.7	-0.9	-0.6
uridine	0.5	0.2	-0.1	0.2	-0.4	-0.3	0.1	-0.5	0.4	0.6		0.3			0.3	-0.9	-0.8	-0.8
acetylcholine	0.7	0.7	0.6	0.7	-0.9	-0.7	0.1	0.1	0.5	0.2	0.1	0.2	0.2	-0.7	-0.1	-0.2	-0.7	-0.4
5-HIAA	0.6	0.4	0.4	0.7	-0.8	-0.9			0.3		-0.2		0.2	-0.6	-0.3	-0.1	-0.5	-0.4
cAMP	0.8	0.5	0.4	0.5	-0.7	-0.7	0.2	0.4	0.5	0.2	0.1	0.3	0.3	-0.4	-0.1		-0.7	-0.3
oleylcarnitine	0.5	0.3	0.4	0.6	-0.7	-0.6		-0.4	0.1	0.1	-0.3	-0.1	-0.3	-0.3	0.3	-0.5	-0.6	-0.4
6-phosphogluconate	-0.8	-0.8	-0.5	-0.3	0.5	0.1	-0.3	-0.3	-0.6	-0.6	-0.4	-0.4	-0.2	0.4	-0.3	0.4	0.7	0.1
adipate	-0.6	-0.3	0.1	0.1	0.4	0.2		0.1	-0.4	-0.6	-0.2	-0.3			-0.6	0.7	0.8	0.3
C18:0 SM	-0.6	-0.2	-0.1	-0.3	0.2	0.3	-0.5		-0.5	-0.7		-0.3	-0.1	-0.3		0.5	0.7	0.8
C16:0 LPC	-0.2	-0.3			-0.1	-0.3	-0.6	-0.2	-0.6	-0.6	-0.6	-0.6	-0.7		0.7	0.2	0.2	0.5
C20:3 LPC	-0.2	-0.4		-0.1		-0.2	-0.5	-0.3	-0.6	-0.6	-0.7	-0.7	-0.8	0.2	0.8	0.1	0.2	0.5
alpha-hydroxybutyrate	-0.1	-0.4	-0.4	-0.1	-0.1	-0.2	-0.4	-0.4	-0.6	-0.4	-0.6	-0.6	-0.7	0.3	0.6	-0.2		0.4
kynurenine	-0.2	-0.7	-0.3		-0.1	-0.4	-0.4	-0.8	-0.7	-0.5	-0.9	-0.8	-0.9	0.5	0.6	-0.3		
C18:0 LPC		-0.4		0.1	-0.1	-0.3	-0.3	-0.3	-0.6	-0.5	-0.7	-0.7	-0.8	0.3	0.8			0.3
inosine		-0.4	0.2	0.1	0.1	-0.2	-0.1	-0.2	-0.5	-0.3	-0.7	-0.6	-0.7	0.5	0.7	0.1	0.1	0.1
C18:2 LPC	-0.2	-0.4	0.2	0.3	-0.1	-0.5	-0.3	-0.2	-0.7	-0.7	-0.8	-0.8	-0.8	0.2	0.4	0.4	0.3	0.4
C16:0 CE	-0.2	-0.4	0.3	0.3	0.3	-0.2	0.1	-0.2	-0.7	-0.4	-0.8	-0.7	-0.8	0.5	0.4	0.3	0.3	0.1
oxalate		-0.1	0.4	0.6	-0.4	-0.6	-0.2	-0.3	-0.6	-0.7	-0.8	-0.8	-0.7	-0.2	0.3	0.2	0.1	0.4
C18:1 LPC		-0.1	0.2	0.3	-0.4	-0.6	-0.5		-0.5	-0.7	-0.6	-0.6	-0.5	-0.2	0.4	0.3	0.1	0.6
C56:8 TAG		-0.2	0.3	0.3	-0.3	-0.6	-0.4		-0.5	-0.7	-0.6	-0.6	-0.5	-0.1	0.4	0.4	0.2	0.5
C54:6 TAG	0.2		0.1	0.4	-0.6	-0.6	-0.3	-0.4	-0.4	-0.4	-0.6	-0.6	-0.6	-0.2	0.6	-0.2	-0.3	0.2
C54:7 TAG	0.3	-0.1	0.2	0.3	-0.3	-0.5	-0.1	-0.3	-0.4	-0.3	-0.6	-0.6	-0.7	0.2	0.7	-0.2	-0.3	0.2
C54:5 TAG	0.3	0.1	0.4	0.6	-0.5	-0.6		-0.1	-0.4	-0.4	-0.6	-0.6	-0.7	-0.1	0.5	0.1	-0.2	0.3
C56:5 TAG	0.3	0.2	0.6	0.7	-0.5	-0.6	0.1	-0.1	-0.4	-0.4	-0.5	-0.6	-0.6	-0.2	0.4	0.1	-0.1	0.3
C56:7 TAG	0.2	-0.2	0.1	0.6	-0.6	-0.9	-0.3	-0.4	-0.3	-0.4	-0.7	-0.6	-0.6	-0.1	0.3	-0.1	-0.3	-0.1
C38:4 PC	0.3	0.1	0.5	0.6	-0.6	-0.8	-0.2	-0.2	-0.2	-0.4	-0.6	-0.5	-0.5	-0.3	0.3		-0.3	-0.1
C58:6 TAG	0.4	0.1	0.5	0.7	-0.5	-0.7	0.1	-0.3	-0.2	-0.2	-0.6	-0.5	-0.6	-0.1	0.5	-0.2	-0.4	
C58:8 TAG	0.5	0.2	0.5	0.7	-0.5	-0.7	0.1	-0.2	-0.2	-0.2	-0.6	-0.5	-0.6	-0.1	0.5	-0.1	-0.4	0.1
C58:7 TAG	0.6	0.3	0.5	0.7	-0.7	-0.8	0.1		-0.1	-0.1	-0.5	-0.3	-0.4	-0.3	0.4	-0.1	-0.5	

Correlation: -1.0 -0.8 -0.6 -0.4 -0.2 0 0.2 0.4 0.6 0.8 1.0

B

Potential metabolic regulators of apoptosis

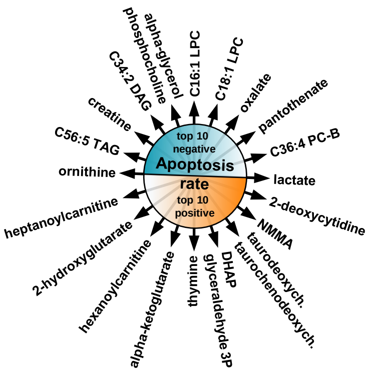

C

Potential metabolic regulators of MMP

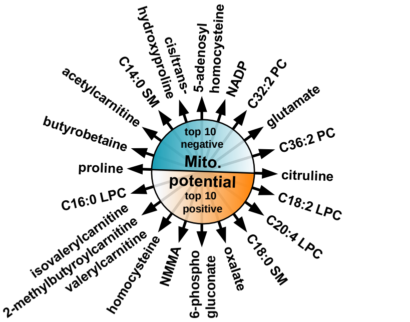

D

Potential metabolic regulators of proliferation

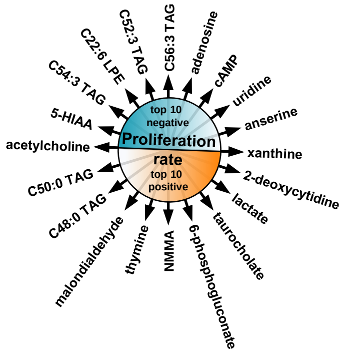

Supplementary Figure 16. Correlating Changes in Drug Response Parameters and Metabolite Levels. (**A**) Correlation analysis linking levels of metabolites and change in cell number upon treatment with a particular compound. Only metabolites displaying simultaneous moderate correlations (<-0.55 or > 0.55) to at least four of the treatments are depicted (67/225). (**B-D**) Schematic representations of metabolites displaying the highest amount of strong correlations whose abundance strongly correlates with apoptosis, MMP and proliferation. Only metabolites displaying the highest amount of strong correlations (minimum 6 correlations; <-0.55 or > 0.55) are displayed. Each scheme depicts 20 metabolites. The abundance of metabolites at the bottom positively correlates with the drug response parameter. Conversely, the abundance of metabolites at the top negatively correlates with the drug response parameter.

Legends for data files S1 to S7

Data S1: Transcriptomic data detailing gene expression in CRC cell lines, extracted from the Broad DepMap Portal and CCLE project.

Data S2: Normalized and transformed LFQ values indicating the abundance of mitochondrial proteins in mitochondria isolated from CRC cell lines.

Data S3: Enrichment analysis of differentially expressed proteins isolated from the mitochondria of CRC cell lines. Fold enrichment scores across all comparisons for each cell line were combined. Similar terms from different libraries were grouped, and fold enrichment scores were converted into Z-scores. An average Z-score was then calculated to determine a consensus enrichment score, which represents the enrichment strength for a specific biological process by integrating information from multiple databases. Differences in consensus enrichment scores between SW480 and SW620 cell lines are highlighted as a subtraction delta.

Data S4: Transformed original values and manually scored values representing the results of bioenergetic and mitochondrial phenotyping of CRC cell lines, integrating cellular features such as volume and proliferation.

Data S5: Metabolomics data detailing the abundance of metabolites in CRC cell lines, extracted from the Broad DepMap Portal and CCLE project.

Data S6: Analysis of correlations between steady-state mitochondrial/bioenergetic characteristics of CRC cell lines and drug response parameters.

Data S7: Analysis of correlations between steady-state metabolite levels in CRC cell lines and drug response parameters. This file also includes correlation values showing the linkage between the abundance of metabolites and the average change in cell number upon treatment with 18 compounds.
