# Supplementary material for: Mitochondrial, metabolic and bioenergetic adaptations drive plasticity of colorectal cancer cells and shape their chemosensitivity
Source: Cell Death Dis. 2025 Apr 5;16(1):253. doi: 10.1038/s41419-025-07596-y (PMC11971274; doi:10.1038/s41419-025-07596-y)
Supplement: Supplementary file 9 — original immunoblots [file 41419_2025_7596_MOESM9_ESM.pdf]

The same membrane but different exposure time

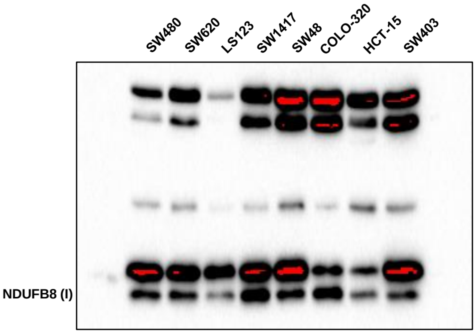

Total OXPHOS Human WB Antibody Cocktail (ab110411)

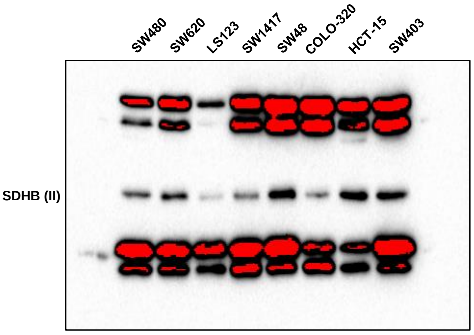

Total OXPHOS Human WB Antibody Cocktail (ab110411)

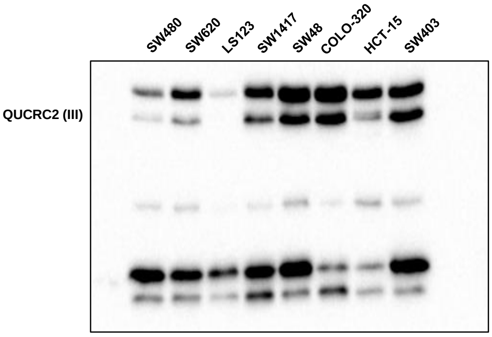

Total OXPHOS Human WB Antibody Cocktail (ab110411)

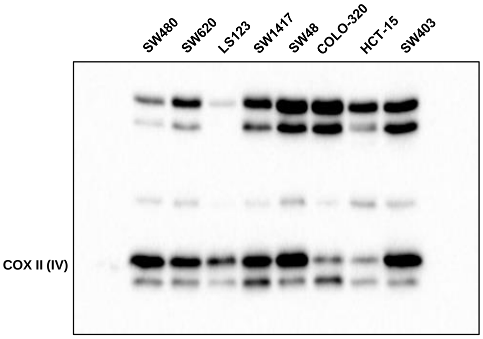

Total OXPHOS Human WB Antibody Cocktail (ab110411)

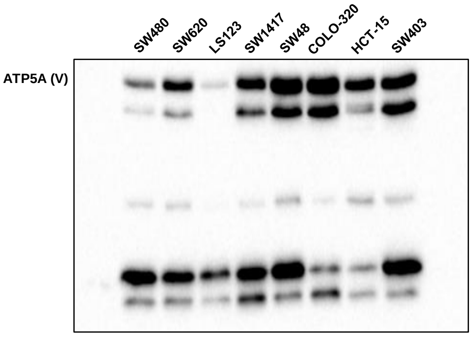

Total OXPHOS Human WB Antibody Cocktail (ab110411)

The same membrane after stripping

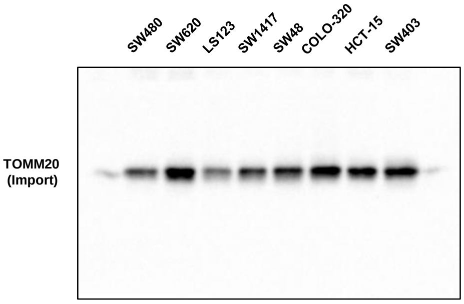

TOMM20 (ab186735)

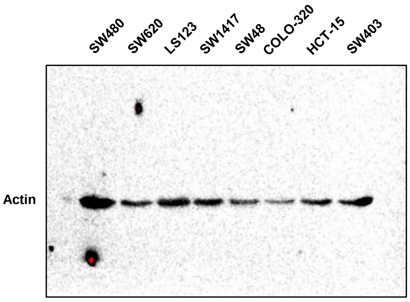

Beta actin (A00730)
